# P-glycoprotein (ABCB1) - weak dipolar interactions provide the key to understanding allocrite recognition, binding, and transport

**DOI:** 10.20517/cdr.2022.59

**Published:** 2023-01-01

**Authors:** Anna Seelig, Xiaochun Li-Blatter

**Affiliations:** Biozentrum, University of Basel, Basel 4056, Switzerland.

**Keywords:** Catalytic cycle, hydrogen bond, π-electron donor, polyspecificity, amphiphilicity, stoichiometry

## Abstract

P-glycoprotein (ABCB1) is the first discovered mammalian member of the large family of ATP binding cassette (ABC) transporters. It facilitates the movement of compounds (called allocrites) across membranes, using the energy of ATP binding and hydrolysis. Here, we review the thermodynamics of allocrite binding and the kinetics of ATP hydrolysis by ABCB1. In combination with our previous molecular dynamics simulations, these data lead to a new model for allocrite transport by ABCB1. In contrast to previous models, we take into account that the transporter was evolutionarily optimized to operate within a membrane, which dictates the nature of interactions. Hydrophobic interactions drive lipid-water partitioning of allocrites, the transport process’s first step. Weak dipolar interactions (including hydrogen bonding, π-π stacking, and π-cation interactions) drive allocrite recognition, binding, and transport by ABCB1 within the membrane. Increasing the lateral membrane packing density reduces allocrite partitioning but enhances dipolar interactions between allocrites and ABCB1. Allocrite flopping (or reorientation of the polar part towards the extracellular aqueous phase) occurs after hydrolysis of one ATP molecule and opening of ABCB1 at the extracellular side. Rebinding of ATP re-closes the transporter at the extracellular side and expels the potentially remaining allocrite into the membrane. The high sensitivity of the steady-state ATP hydrolysis rate to the nature and number of dipolar interactions, as well as to the dielectric constant of the membrane, points to a flopping process, which occurs to a large extent at the membrane-transporter interface. The proposed unidirectional ABCB1 transport cycle, driven by weak dipolar interactions, is consistent with membrane biophysics.

## INTRODUCTION

P-glycoprotein (ABCB1) (170 kDa) was discovered in 1976 by Juliano and Ling^[1]^ in Chinese hamster ovary (CHO) cells, selected for resistance to colchicine. These cells displayed *pleiotropic cross-resistance* to a wide range of *amphiphilic* drugs. Because the glycoprotein altered the *membrane permeability (P)*, it was called P-glycoprotein. This first description comprises the key characteristics of the protein (highlighted in italics), which will play a recurrent role in this review. ABCB1 is the first mammalian member of a large family of ATP binding cassette (ABC) transporters present in prokaryotes^[2]^ and eukaryotes, from plants^[3]^ to humans^[4]^. Most ABC transporters move compounds (allocrites^[5]^) across membranes, using the energy of ATP binding and hydrolysis. The functional unit of ABC transporters consists of two highly conserved nucleotide-binding domains (NBDs) forming together two nucleotide-binding sites (NBSs) for ATP hydrolysis and two more variable transmembrane domains (TMDs), which are assumed to form the translocation pathway for allocrites. In prokaryotes, the functional unit consists of a homodimer formed by two polypeptides with one NBD and one TMD each. In most eukaryotes, these two polypeptides are linked and the transporters function as monomers. In recent years, approximately 250 structures of ABC transporters have been obtained, allowing the establishment of a new classification system based on TMD folds^[6]^. Prokaryotic ABC transporters revealed seven types of folds (I-VII), while eukaryotic ABC transporters had only two (IV and V). The seven subfamilies (ABCA-G), defined previously based on phylogenetic analysis^[7]^, are maintained as subcategories within the type IV fold (subfamilies B-D) and type V fold (subfamilies A and G). Although the large number of structural approaches have provided important information, “the question as to how substrate binding and translocation are coordinated and coupled with ATP binding and hydrolysis, in any ABC transporter, remains elusive”^[8,9]^.

Several models for ABCB1 function have been suggested. Senior and colleagues^[10]^ observed that both NBSs of ABCB1 were catalytically active and seemed to alternate in activity, hydrolyzing one ATP per catalytic cycle with a turnover of 1-10 molecules s^-1^. They suggested a scheme of alternating catalytic sites, in which drug transport is coupled to the relaxation of a high-energy catalytic site conformation, generated by the hydrolysis step^[10]^. Experiments by Sharom and colleagues^[11]^ supported this model (for review, see^[12]^). More recently, one study demonstrated that ABCB1 “mutants with one intact catalytic center preserve the ability to hydrolyze ATP and to promote drug transport, suggesting that the two catalytic sites are randomly recruited for ATP hydrolysis”^[13]^. An analogous observation was made for ABCC7 (cystic fibrosis transmembrane conductance regulator, CFTR) that lacks the catalytic glutamine in NBD1^[14]^. Ambudkar and Sauna suggested that transport is coupled to the hydrolysis of a first ATP and resetting of the transporter to the hydrolysis of a second ATP^[15]^.

On the basis of the first low-resolution structures of ABCB1 in the absence and presence of nucleotides^[16]^, Higgins and Linton proposed the currently prevailing “ATP switch model”^[17]^. This model assumes that binding of two ATP molecules induces an outward-facing (OF) conformation for drug release, and hydrolysis of two ATP molecules leads to a nucleotide free (apo-form) with an inward-facing (IF) conformation for drug binding. Support for this switch, or alternating access model, was inferred from the OF high-resolution conformation of the homodimeric Sav1866 in the presence of two AMP-PNP molecules^[18]^, and the IF conformation of apo-ABCB1^[19,20]^. An IF conformation of ABCB1 was also observed in permeabilized, ATP-depleted cells, in the presence of the conformation-sensitive antibody UIC2 mAb^[13]^. The resemblance of the alternating access model to the early “simple allosteric model for membrane pumps” by Jardetzky^[21]^ proposed for moving inorganic ions across membranes was taken as further support for this model (for review, see e.g.,^[22,23]^).

The currently prevailing “alternating switch model” raises several questions. (i) Is the aqueous cleft of the apo-state, wide open to the cytosol, useful for the transport of amphiphilic compounds that highly accumulate in membranes and access the transporter within the membrane^[24-27]^? (ii) Is the assumption of an apo ground state realistic, considering the high intracellular ATP concentration (c_ATP_ = 1-10 mM)^[28-30]^ and the comparatively low *K*_m_ values for ATP binding to the transporter (c_ATP_ = 0.4-0.8 mM)^[31,32]^? Are two ATPs hydrolyzed, or is one ATP hydrolyzed per transport cycle?

To obtain information on the conformation of the NBDs and TMDs under turnover conditions, we analyzed the results of multiple double electron-electron resonance (DEER) spectroscopy experiments performed with spin label pairs introduced at strategic locations in different ABC transporters and compared them with X-ray structures. The DEER experiments revealed a wide range of conformations that were not fully accounted for in the proposed models (see Ref.^[33] ^and Supplementary Table 1 therein).

For further insight, we performed molecular dynamics (MD) simulations for different nucleotide occupancy states of Sav1866 structure, a prokaryotic ABCB1 homolog^[33]^. The two transporters (both with a type IV fold^[6]^) show overlapping substrate specificity^[32,34]^. Our simulations revealed an outward closed conformation of the transmembrane domain that is stabilized by the binding of two ATP molecules. The hydrolysis of a single ATP leads the X-loop, a key motif of the ATP binding cassette, to interfere with the transmembrane domain and favors its outward open conformation. These findings provided a structural basis for the unidirectionality of transport in ABC exporters and suggested that one ATP is hydrolyzed per transport cycle^[33]^. However, the role of the amphiphilic allocrites in the transport process remained unclear.

Here, we demonstrate that a quantitative understanding of the interactions between the amphiphilic allocrites and the transporter is possible if the membrane environment is taken into account. For this purpose, we review the thermodynamics (allocrite binding) and kinetics (ATP hydrolysis and allocrite transport rate) of ABCB1, published over approximately the past 25 years. Altogether, this work provides a compelling quantitative description of the nature of the intermolecular interactions relevant for ABCB1 function: Allocrite binding within the membrane, the rate of ATP hydrolysis, and allocrite transport are driven by weak dipolar forces that depend on the nature of the surrounding membrane. Combining the insights gained with the conclusions from our previous molecular dynamics simulations^[33]^ generates a unidirectional allocrite transport cycle for ABCB1 that is consistent with the principles of membrane biophysics. Special emphasis is placed on clarifying the mechanism of ABCB1 inhibition, which is of crucial relevance for pharmacotherapy.

## THE REACTION PARTNERS AND THEIR ENVIRONMENT

Understanding chemical processes generally starts with structural analysis of the reactants, in the present case, the ABCB1 transporter and its allocrites. Allocrites include substrates, modulators, and inhibitors (for definitions used, see Ref.^[35]^). Although less recognized, the environment in which the reaction partners meet determines the nature of interactions. Because ABCB1 and its allocrites meet in the membrane, we first provide a short description of the lipid bilayer and its properties relevant for the subsequent discussion of allocrite-transporter interactions (Supplementary Equation 1, discussed in detail below).

### Characteristics of lipid membranes relevant for allocrite-transporter interactions

ABCB1 is abundant in plasma membranes. The plasma membrane shows an asymmetric lipid distribution between the bilayer leaflets. The extracellular leaflet is composed essentially of electrically neutral lipids (phosphatidylcholines, sphingomyelins, and cholesterol). The cytosolic leaflet consists of electrically neutral phosphatidylethanolamines and anionic phosphatidylserines (for details, see^[36,37]^). The cytoplasmic leaflet is approximately twofold more unsaturated than the extracellular leaflet. These structural asymmetries are conserved throughout eukaryotes, suggesting fundamental cellular design principles^[37]^.

The membrane is well described by its molecular order parameter, S_mol_, the lateral packing density, π_M_, the surface potential, *Ψ*_m_, and the dielectric constant, ε_m_. Each parameter is briefly explained below.

#### Molecular order parameter, S_mol_, of lipid bilayers in the presence of “guest” molecules

Although membranes are highly organized, they are recalcitrant to crystallization because of considerable translational, rotational, and flexing movements of the constituent lipid and protein molecules. The dynamic “structure” of biological membranes is best characterized by deuterium nuclear magnetic resonance (^2^H- or D-NMR) spectroscopy^[38,39]^. Chemically or biochemically exchanging protons with deuterons, either in the polar head group or in the acyl chains of lipids, provide information on the order and mobility of the molecules without disturbing the system. This is in contrast to most other labels including spin labels or fluorescent labels such as DPH (trimethylamine-diphenylhexatriene). Below, we give a few representative examples of how “guest” molecules (including cholesterol, peptides, membrane proteins, detergents, and drugs) influence the molecular order parameter of phospholipid bilayers.

#### Cholesterol

The addition of cholesterol to a dipalmitoylphosphatidylcholine (DPPC) bilayer in an equimolar amount doubles the order parameter of the fatty acyl chain region and eliminates the gel-to-liquid crystal phase transition (phase transition temperature for DPPC, T_m_ = 41 °C), producing a smooth order-temperature profile^[40]^. An enhanced order parameter was also reported for bilayers by 1-palmitoyl-2-oleoyl-sn-glycerol-3-phosphocholine (POPC, with T_m_ = -2 °C)^[41]^.

#### Peptides and proteins

The interaction of transmembrane proteins such as cytochrome c oxidase with the surrounding lipids has been investigated extensively by spin-label electron paramagnetic resonance (EPR) and NMR spectroscopy. EPR measurements (monitoring a high frequency range ~10^7^-10^8^ Hz) revealed two motionally distinct lipid populations. The term “boundary lipids” was coined for the slower component^[42]^. However, subsequent ^2^H-, ^31^P-, and ^14^N-NMR investigations (monitoring a frequency range at least 10-fold lower) did not detect the presence of two lipid populations^[43-45]^. All lipids around the reconstituted cytochrome c oxidase exhibited very similar motional behavior and provided no evidence for motionally restricted boundary lipids, neither at the head group region nor at the cis-double bond^[46]^. To investigate potential interactions between cytochrome c oxidase and specific lipids, we characterized the few residual lipids attached to cytochrome c oxidase after delipidation using ^31^P-NMR spectroscopy^[47]^. While most previous biochemical studies claimed that cardiolipin was the only lipid remaining after delipidation, the ^31^P-NMR data show that all three lipids (phosphatidylethanolamine, phosphatidylcholine, and cardiolipin) can be observed, with cardiolipin being the least abundant. Compared with the effect of cholesterol, the effect of proteins and peptides on lipid membranes is thus generally almost negligible, indicating a perfect match between the movement of fluid-like hydrocarbon chains and the movement of the peptide side chains^[48,49]^. Natural cells also revealed rapid exchange of lipids around proteins on the NMR timescale (frequency range ~10^6 ^Hz)^[50]^.

#### Detergents and drugs

Detergents (e.g.,^[51,52]^) and drugs (e.g.,^[53]^) have been well documented to cause membrane disorder at higher concentrations. Many detergents and drugs are allocrites for ABCB1. Interestingly, ABCB1 exports these drugs and detergents well below the concentrations that lead to significant disorder of membranes (see below).

#### Mixing disparate lipids induces domain formation

Mixing bulky disordered lipids, such as the non-physiological DOPC or fluorescent lipids, with highly ordered lipids such as cholesterol and DPPC (e.g.,^[54]^) readily induces domain formation or phase separation. Hell and coworkers observed that, unlike phosphoglycerolipids, fluorescently labeled sphingolipids and glycosylphosphatidylinositol-anchored proteins can be transiently trapped (for approximately 10-20 ms) in cholesterol-mediated molecular complexes dwelling within < 20-nm-diameter areas^[55]^.

#### Lateral membrane packing density, π_M_

Generally, a higher order parameter S_mol_ is associated with higher lateral packing density of the membrane, π_M_. A model membrane consisting of POPC, which is the most abundant lipid in mammalian membranes, exhibits a lateral packing density π_M_ ≈ 32 mN/m at room temperature^[56]^. The addition of cholesterol (25 mol %) enhances the lateral packing density to π_M_ ≈ 35 mN/m^[57]^. The lateral packing density, π_M_, is relevant because it determines partitioning of compounds into the membrane^[58]^. To give an example embryonic cells (e.g., mouse embryo fibroblasts, NIH-3T3 cells^[25]^), with a low cholesterol content, exhibit lower lateral packing densities than adult mammalian cells^[59]^.

#### Surface potential, Ψ_m_

The cytoplasmic membrane leaflet exhibits a negative surface potential, *Ψ*_m_. For mouse embryo fibroblasts (NIH-3T3 cells^[25]^), it was estimated as *Ψ*_m_ ≈ -30 to -20 mV under physiological conditions, assuming a cytosolic free magnesium concentration of C = 0.5 to 1 mM (with a membrane-binding constant for magnesium, *K* = 10 M^-1^) and a monovalent cation concentration of C = 100 to 150 mM (with a membrane-binding constant for monovalent cations, *K* = 0.6 M^-1^)^[59]^. Under the given conditions, a surface potential of *Ψ*_m_ ≈ -30 mV enhances the lipid-water partition coefficient, *K*_lw_, of a strongly cationic drug by a factor of ~4. However, upon titration with cationic drugs, the surface potential decreases (see Ref.^[32]^, Figure 7 therein).

Owing to the negative surface potential and the high unsaturation, the cytosolic plasma membrane leaflet acts as a drug scavenger for amphiphilic and cationic ABCB1 allocrites. The properties of the cytosolic membrane leaflet may even create a drug concentration gradient within the membrane that is opposed to the concentration gradient in the extracellular *vs.* intracellular aqueous phase. Notably, model membranes generally lack the asymmetry of natural membranes.

#### The dielectric constant, ε_m_

Plasma membranes separate the extracellular and intracellular aqueous phases. Whereas the aqueous phase exhibits a high dielectric constant (ε_m_ ≈ 80), the polar lipid head group regions of the membrane exhibit an intermediate one (ε_m_ ≈ 30-40) and the hydrophobic core region a very low one (ε_m_ ≈ 2-4)^[60,61]^. The dielectric constant*,* ε_m_, decreases with increasing lateral membrane packing density, π_M_, and thus varies somewhat with the lipid chain length, the degree of unsaturation, the cholesterol content, and the phase state of the membrane. The low dielectric constant, ε_m_, of the membrane strengthens dipolar interactions.

### Allocrite recognition

**Figure 1:**
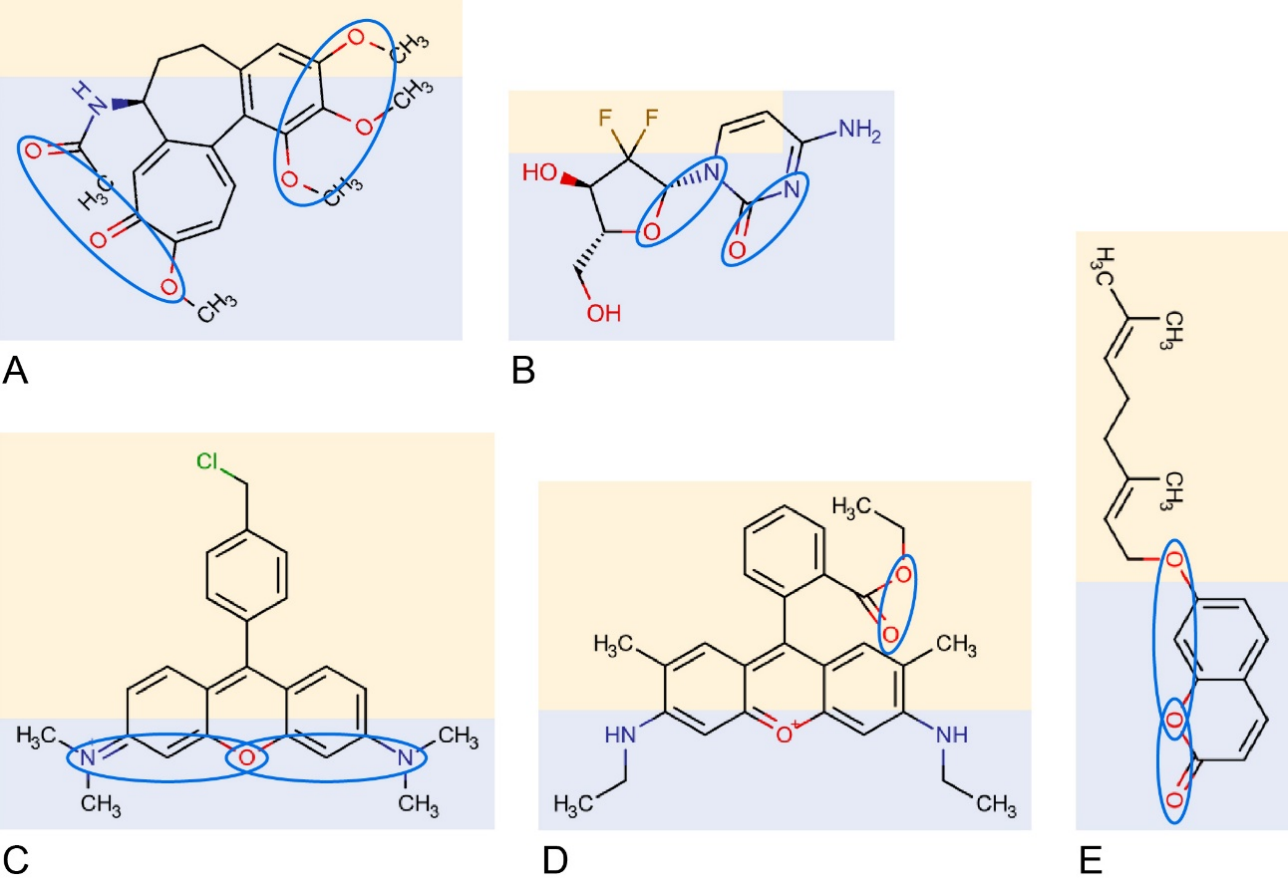
Examples of allocrites. Allocrites for ABCB1 are amphiphilic (polar part in blue, hydrophobic part in yellow) and carry type I or type II π-electron donor patterns (i.e., hydrogen bond acceptor patterns, HBAs) that are attracted by the HBDs in the protein. A type I pattern contains two HBAs separated by 2.5 ± 0.3 Å and a type II pattern contains two or three HBAs, where the outer two are separated by 4.5 ± 0.6 Å. Possible type I and type II patterns are encircled in blue: (A) *Colchicine*, two type II patterns. (B) *Gemcitabine* is an anticancer drug, which induces cell death by blocking DNA replication, with either two type I patterns (shown) or one type II pattern (not shown). (C)* Tetramethylrosamine* (*TMR*), type II patterns. (D) *Rhodamine 6G* (*R6G*), one type I pattern (secondary and primary amino groups are not involved in patterns). (E) *Auraptene*, a citrus phytochemical, one type I or one type II pattern. The orientation of the π-electrons in a pattern does not seem crucial. Unsaturated rings play a role in π-π stacking interactions.

Long before ABCB1 structures were available, hundreds of allocrites had been identified. Allocrites are amphiphilic (or amphipathic)^[1]^ and often cationic^[62]^. To explain the “polyspecificity” of ABCB1, we searched for recurrent elements in the chemical structures of drugs with the ability to interact with the transporter *within* a lipid environment^[24-27]^ (see also Ref.^[63]^). The analysis of a large number of chemical structures revealed specific patterns formed by *Π-*electron donor groups, that is, hydrogen bond acceptor groups (HBAs) and *Π-*electron systems (i.e., aromatic rings)^[64,65]^ (see also^[66,67]^). The assumption that these patterns interact with the hydrogen bond donor groups (HBDs) and *Π-*electron systems (phenyl and tryptophan residues) in transmembrane helices of ABCB1 through dipolar interactions offers an explanation for the polyspecificity of ABCB1. Figure 1A-E display compounds with possible type I *and/or* type II patterns. Compounds with type II patterns are not only allocrites for ABCB1 but also inducers of ABCB1 expression by interacting with the nuclear pregnane X receptor, for example^[68]^.

Notably, HBDs in allocrites (e.g., -OH, -NH_2_, and > NH) do *not* interact with ABCB1^[64]^. However, they significantly reduce the lipid-water partition coefficient (e.g., Refs.^[48,69]^) and the rate of passive diffusion across the lipid membrane, which in turn enhances the risk of drugs being caught by ABCB1^[58]^.

### The transporter exposes multiple HBDs

**Figure 2:**
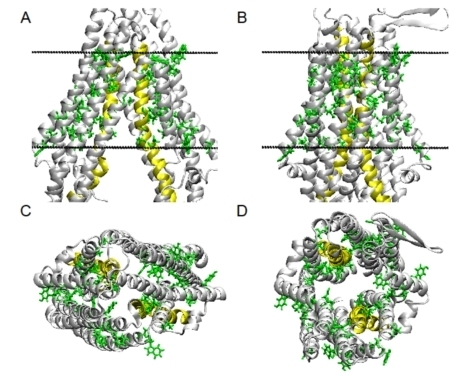
Amino acid residues with HBD side chains of ABCB1 at the level of the membrane (NBDs are truncated). (A) Abcb1a structure of the apo conformation open to the cytosol (PDB entry 4m1m) (side view). (B) Model of the closed conformation, based on the crystal structure of Sav1866 with two nucleotides bound (PDB entry 2hyd) (side view). (C) Apo conformation (top view). (D) Model of the closed conformation (top view). Amino acid side chains with HBDs are shown. TMD6 and TMD12 are colored yellow, whereas other helices are colored light gray. The two dashed lines indicate the position of the membrane (adapted from Ref.^[67]^, for details see Supplementary Figure 1A and B).

Figure 2 (and Supplementary Figure 1A-D) displays the numerous HBDs in the transmembrane domain of apo-Abcb1a^[20]^ and the nucleotide-bound ABCB1 (modeled on the high-resolution structure of Sav1866^[18]^) from side [Figure 2A and B] and top views [Figure 2C and D]. The amino acids with hydrogen bond donor groups are highlighted in green. Interestingly, many HBDs are oriented towards the lipid phase. The HBDs in transporters most likely play a dual role; on the one hand, they extract compounds with appropriate HBAs (i.e., allocrites) from the lipid membrane, and on the other, they allow allocrite gliding across the membrane^[67]^. In this context, phenyl residues that can undergo π-π stacking interactions with unsaturated rings in allocrites may also play a role (see Supplementary Figure 2A-D). The homodimeric Sav1866 from *Staphylococcus aureus*^[18]^ is the prototypical type IV fold protein and a homolog of the monomeric Abcb1a and ABCB1. These proteins share the cross-over of helices: in the case of the homodimeric Sav1866, helices 4 and 5 from each monomer cross over to the other monomer; in the case of ABCB1, helices 4 and 5 from TMD1 cross over to the C-terminal TMD2 and helices 10 and 11 from TMD2 cross over to the N-terminal TMD1^[6]^. Because the X-ray structure of Sav1866 was obtained at a high resolution (3.0 Å)^[18]^, it provides an ideal basis for modeling other type IV fold proteins.

### Transporters with allocrites bound - structural insights

In recent years, several atomic structures of ABC transporters with allocrites bound have been resolved. Most of them are in the apo form. The first crystal structure of ABCB1 (at 4.5-3.8 Å resolution) was obtained with the hydrophobic cyclic peptides QZ59-RRR and QZ59-SSS bound to the transmembrane domain. The peptides and the transporter were assumed to connect via “hydrophobic” interactions^[19]^. Because the peptides carry several weak type I patterns formed by nitrogen and selenium, hydrogen bonding with the transporter is possible. The yeast mitochondrial ABC transporter Atm1 (an ortholog of human ABCB7) was crystalized (at 3.06 Å and 3.38 Å resolution) with the substrate glutathione. It revealed hydrogen bond formation between allocrite and transporter^[70]^. Locher and colleagues provided several atomic structures (at 4.0-3.2 Å resolution) obtained by cryo-electron microscopy (EM) of human ABCB1 with the three inhibitors elacridar, tariquidar, and zosuquidar, or vincristine bound^[71]^. The allocrites are surrounded by ample amino acids with π-electron systems or hydrogen-bonding groups (see, e.g., vincristine^[71]^ or taxol^[72]^). The above inhibitors appear as pairs, arranged either in sequence (one behind the other) or in parallel or antiparallel orientation, respectively (see Ref.^[71]^, Supplementary Figure 8 therein). Similar observations were made with two molecules of encequidar bound to ABCB1^[73]^.

Similar to lipid membranes, transmembrane proteins are highly flexible and cannot be crystalized, unless they are stabilized. Molecules that may be favorable in this respect are dodecylmaltoside (DDM) that is an inhibitory detergent, inhibitory allocrites (see below), or the conformation-sensitive antibody UIC2 mAb. The variation of crystal contacts under different crystallization conditions may also play a role in the wide distribution of conformations observed in ABC transporters^[18,74,75]^. Even if atomic structures may not reflect the functionally relevant conformations, they provide relevant aspects, such as the long predicted (i) broad binding areas that can accommodate two allocrites simultaneously (^[76,77] ^see below); and (ii) the allocrite binding mode via weak dipolar interactions^[64,65]^.

## ALLOCRITE BINDING TO ABCB1 - A TWO-STEP PROCESS

Allocrite binding from water to the transporter characterized by the transporter-water binding constant *K*_tw_ occurs in two steps. The first step is allocrite partitioning from the aqueous phase into the lipid membrane, characterized by the lipid-water partition coefficient, *K*_1w _(M^-1^). The second step is allocrite attraction to the transporter within the membrane, characterized by the transporter-lipid binding constant *K*_tl _(dimensionless). The transporter-water binding constant *K*_tw _(M^-1^) can therefore be expressed as the product of the partition coefficient *K*_lw_ and the binding constant *K*_tl_ within the membrane [Supplementary Equation 1].

For simplification, we use free energies of binding in the following instead of binding constants. They can be interpreted as affinities and are additive [Supplementary Equations 2-5]. The free energy of allocrite binding from water to the transporter, 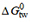, is the sum of the free energy of partitioning into the membrane 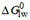 (step I) and the free energy of binding to the transporter within the membrane 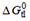 (step II) [i.e., 
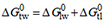
, (Supplementary Equation 2)]. Methods for assessing the binding constants and the free energies of binding are detailed in the Supplementary Materials.

### Quantification of the individual binding steps 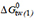, 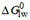, and 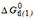


The free energy of binding from water to the activating binding region of the transporter in inside-out plasma membrane vesicles^[78]^ and live NIH-MDR1-G185 cells was determined as 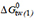 = -30 to -54 kJ mol^-1^, the free energy of allocrite partitioning from water into the lipid membrane as 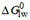 = -23 to -34 kJ mol^-1^, and the free energy of allocrite binding from the lipid membrane to the activating binding region of the transporter as the difference between these two free energies [Supplementary Equation 2], 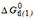 = -7 to -27 kJ mol^-1^. The values given refer to the lowest and highest values for each type of free energy of binding, among the 19 compounds investigated. 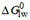 contributes significantly to the overall binding, but the free energy of allocrite binding to the transporter within the membrane 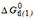 varies more strongly than the free energy of lipid-water partitioning 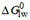.

### Characterization of the driving forces

The partitioning of uncharged amphiphiles such as drugs and detergents into electrically neutral membranes is driven by hydrophobic interactions. The classical hydrophobic effect (i.e., the release of ordered water molecules surrounding the solute in the aqueous phase upon partitioning into the membrane) is essentially entropy-driven (increasing with cooling), as observed for cyclosporine A^[48]^ and nonionic detergents^[79]^, for example. Partitioning of electrically charged drugs into an electrically neutral membrane is best described by the “non-classical hydrophobic effect”, which is essentially enthalpy-driven (increasing with warming)^[80]^. The same is true for the cationic n-alkyl trimethyl ammonium chlorides (C_m_-TACs) (see Ref.^[79]^, Table 1 therein). Partitioning of amphiphiles decreases exponentially with increasing lateral packing density of the membrane, π_M_, and increasing cross-sectional area of the partitioning amphiphile, A_D _(Supplementary Equations 6 without and 7 and 8 with a negative surface potential, *Ψ*_m_).

**Figure 3:**
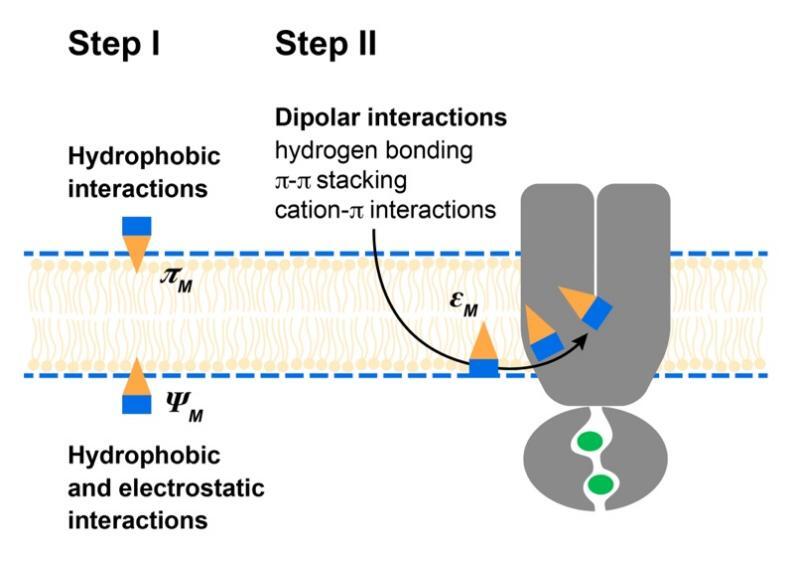
Scheme showing the types of interactions in the two-step process of allocrite binding from the aqueous phase to ABCB1 within the membrane. Step I: Partitioning of an amphiphilic allocrite (with polar part in blue and hydrophobic part in yellow) into the extracellular membrane leaflet depends on the lateral packing density of the membrane, π_m_. Moreover, partitioning into the cytosolic leaflet depends in addition on the surface potential *Ψ*_m_ of the membrane^[59]^. Step II: Dipolar interactions between allocrite and transporter (including hydrogen bonding, π-π stacking, and π-cation interactions, given in the order of assumed relevance) are suggested to drive recognition, binding, and “transport” of the polar part to the middle of the membrane. Thereby it is assumed that the hydrophobic part remains in contact with the lipid environment.

To extract amphiphiles out of the most hydrophobic environment in a cell, that is, the lipid bilayer, hydrophobic interactions are not useful. Amphiphiles such as drugs and detergents can however be extracted by dipolar interactions^[81,82] ^[Figure 3]. An interesting aspect of the nature of dipolar interactions was revealed from a comparison of studies on allocrite binding to ABCB1 in liquid-crystalline and gel-state membranes^[83,84]^. Whereas partitioning of allocrites into the gel-state membrane was lower than into the liquid-crystalline membrane, as expected, because of the enhanced lateral packing density in the gel-state membrane [Supplementary Equation 6^[58]^], binding to the transporter within the gel-state membrane increased two to fourfold^[84]^. The two to fourfold increase in the binding constant to the transporter *K*_tl_ within the gel-phase membrane is consistent with a slight decrease of the dielectric constant ε_m_ in the gel phase as compared with that in the liquid-crystalline phase (see e.g., Ref.^[61]^) [Supplementary Equations 9-11].

Thus, partitioning into the membrane is driven by hydrophobic interactions and limited by the lateral membrane packing density π_m_, whereas allocrite binding to the transporter within the membrane is driven exclusively by dipolar interactions that increase with increasing membrane packing density π_m_ and concomitantly decreasing dielectric constant ε_m_.

## ATP HYDROLYSIS IN INSIDE-OUT PLASMA MEMBRANE VESICLES

### Early titrations of ABCB1 with drugs yielded bell-shaped activity curves

**Figure 4:**
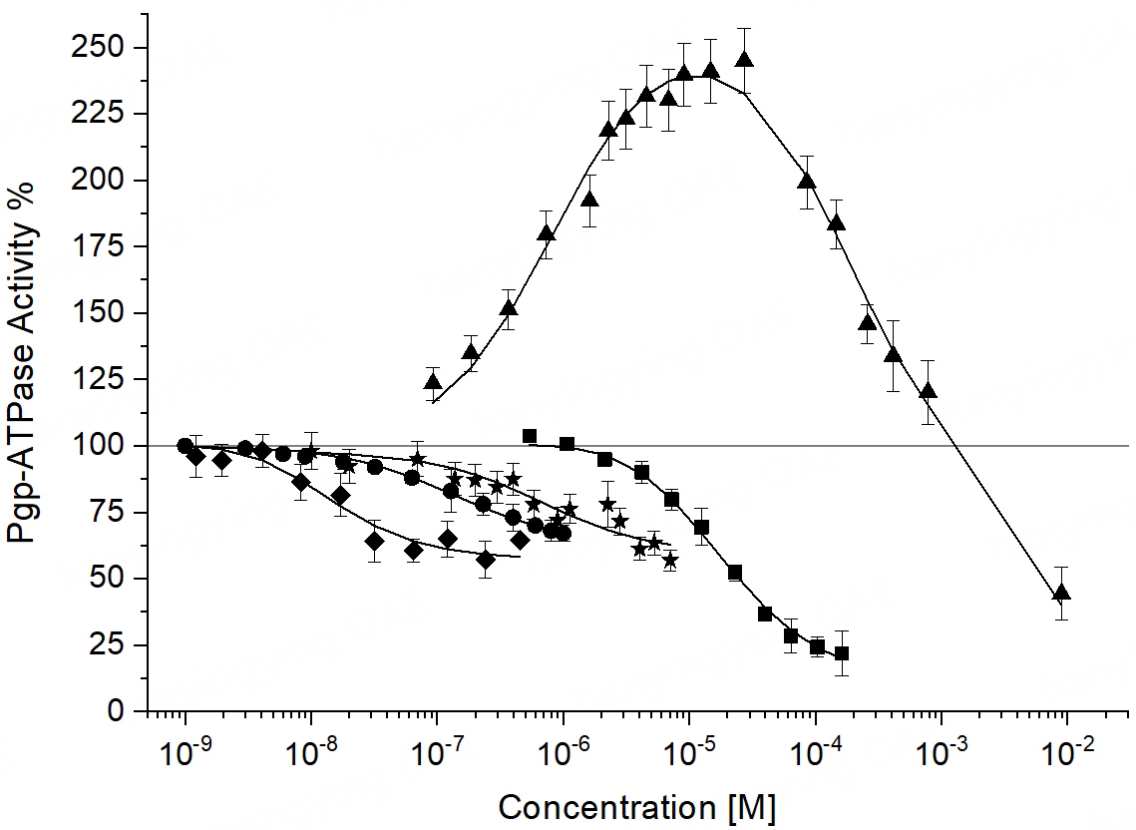
ABCB1 ATPase activity *vs.* allocrite concentration curves. ATPase activity measured in inside-out plasma membrane vesicles from NIH-MDR1-G185 cells^[25]^. Data are fitted to the two-site binding model by Litman *et al.*^[96]^. Tariquidar (diamonds); OC144 093 (circles), cyclosporine A (stars), DDM (squares), verapamil (triangles). Tariquidar, OC144 093, and verapamil inhibit as dimers. DDM^[79]^ and cyclosporine A (see Ref.^[79]^, Supplementary Table 3 therein) most likely inhibit as monomers due to their unfavorable *q* = 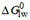/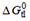 values (see below).

Monitoring ATP hydrolysis as a function of drug concentration was, and still is, the key experiment for understanding how ABC transporters catalyze allocrite transport or flopping. The first titrations of ABCB1 with drugs were performed with inside-out plasma membrane vesicles. As they expose the NBDs to the extravesicular aqueous phase, the release of inorganic phosphate during ATP hydrolysis can be easily monitored by spectroscopic techniques (see e.g.,^[76]^). The plasma membranes used originated from various ABCB1-overexpressing cell lines, including mouse embryo fibroblasts^[85]^, ovarian carcinoma cells (2780^AD^)^[86]^, insect cells^[87,88]^, Chinese hamster ovary cells, CHRC5^[89]^, CR1R12^[90]^, CH^r^B30 cells^[91]^, and Ehrlich ascites tumor cells^[76]^. Most early ABCB1 titrations with drugs show a rise in steady-state ATPase hydrolysis at low concentrations up to a maximum, followed by a decrease at high concentrations, yielding characteristic *bell-shaped *activity (or velocity, V) *vs.* concentration curves [Figure 4]. ABCB1 titrations in plasma membrane vesicles with verapamil exhibited maximum steady-state ATP hydrolysis rates (V_max_) around the concentration C_verap_ ≈ 10 μM, even though the membranes originated from different cell lines. This may be due to the subtle compensation between the reduced allocrite partitioning into membranes of higher lateral packing density and the enhanced dipolar affinity between the allocrite and the transporter within the membrane. The drug-stimulated ATPase activity was directly proportional to the amount of P-glycoprotein, as demonstrated in Ehrlich ascites tumor cell lines^[76]^, whereas the concentration of half-maximum activation remained approximately constant. In reconstituted proteoliposomes, the concentration of maximum activity depends on the type and residual concentration of detergents used for reconstitution, as discussed below.

### Bell-shaped ATPase activity curves - artifact or fact?

Around the turn of the millennium, some skepticism arose regarding the inhibitory branch of ABCB1 activity curves and various artifacts were suspected of having influenced the titration curves at high drug concentrations, including membrane disordering, vesicle aggregation, ATP depletion, and a disturbed boundary layer around the protein. To clarify the ABCB1 kinetics, we investigated the potential artifacts.

#### Membrane disordering?

In 1995, Drori *et al.* observed that the cytotoxicity of anticancer drugs in multidrug-resistant cell lines was potentiated by chemosensitizers (i.e., detergents)^[92]^. They hypothesized that chemosensitizer-mediated membrane perturbations could interfere with the ATPase activity of ABCB1 and/or with ABCB1 drug transport capability. We know today that the detergents used by Drori *et al.* are allocrites for ABCB1 and act as ABCB1 inhibitors at higher concentrations^[69,79,81,82,92]^.

To quantify the disordering effect of detergents and drugs at concentrations relevant for ABCB1 activation and inhibition, we measured the order parameter S_mol_ by D-NMR spectroscopy. The effects were negligibly small at concentrations of half-maximum activation, *K*_1_, and showed a reduction in the order parameter of only about 5% in the case of detergents at half-maximum inhibition, *K*_2_^[79]^. Somewhat more disordering was observed in the case of verapamil, owing to its larger cross-sectional area in the folded, membrane-bound conformation^[93]^ (for quantitative data, see Supplementary Table 1 and references therein). Thus, ABCB1 eliminates allocrites before membrane disordering effects become obvious.

Moreover, a transition state analysis of ABCB1 activity in membrane environments exhibiting different lateral packing densities revealed that the transporter acts in a broad range of environments from densely packed lipids to loosely packed micelles (see Ref.^[94]^, Figure 6 therein). With decreasing membrane packing density, the activation energy decreased and thus the rate of ATP hydrolysis increased. Whereas the activity was entropy-driven at a high packing density, it was essentially enthalpy-driven at a low packing density^[94]^. The activity of ABCB1 is thus robust with respect to its environment and it works perfectly in a much broader range of membrane packing densities than encountered under physiological conditions.

#### Vesicle aggregation?

In inside-out plasma membrane vesicles, the negatively charged inner membrane surface is exposed and thus these vesicles repel each other. Upon titration with strongly cationic drugs, the negative surface potential is strongly reduced, which may lead to vesicle aggregation at high concentrations. Even under these conditions, a decrease in activity occurs before aggregation takes place. The phenomenon of vesicle aggregation at high drug concentrations is limited to compounds with pK_a_ > 9. Cationic drugs with lower charge or no charge exhibit no vesicle aggregation, and show bell-shaped curves as well (for details, see^[78]^).

#### ATP depletion?

ATP depletion has been observed with pluronic block copolymers^[95]^ at an incubation time of 2 h. This is at least twice the incubation time generally used in ATPase activity measurements. The titration of ABCB1 with verapamil under ATP regenerating conditions again yielded practically identical bell-shaped activity *vs.* concentration curves (see Ref.^[66]^, Figure 2 therein).

#### Disturbed boundary lipids?

As mentioned above, boundary lipids or lipid domains were not observed in natural membranes at physiological temperatures upon monitoring different deuterated phospholipids by D-NMR spectroscopy^[50]^. Disturbed boundary layers, that is, disordered membranes of lower packing density, would enhance rather than reduce the steady-state ATP hydrolysis rate (for details, see below). We conclude that the decrease in ABCB1 ATPase activity at high drug concentrations is real and highly relevant for understanding transporter inhibition (see below).

### Two-site binding models are required

In 1997, Stein and colleagues proposed a model for quantitative evaluation of bell-shaped ABCB1 activity *vs.* allocrite concentration curves^[76,96]^. This model is based on the principle of Michaelis-Menten kinetics and takes into account basal activity, V_0_, in the absence of an exogenous allocrite; enhanced activity, V_1_, with one allocrite molecule bound to the transporter; and reduced activity, V_2_, with two allocrite molecules bound to the transporter [Supplementary Equation 12] (see Figure 4). A related model, however, assuming basal activity, uncoupled from transport, a drug-activated phase, and a drug-inhibited phase, was later proposed by Al-Shawi and colleagues^[77]^. The recent advances in atomic transporter structures provide direct evidence for *two* molecules bound to ABCB1 (see e.g., Ref.^[71]^) and support the necessity of two-site binding models. We consider the model proposed by Stein and colleagues^[76,96]^ as more plausible because empty cycling seems unlikely from an energetic point of view.

### Allocrites that may contribute to basal activity in plasma membrane vesicles

Basal activity was proposed to be due to an as-yet-unknown allocrite^[10]^. Different endogenous allocrites may be considered. The most prevalent among them is POPC. Protonated POPC (POPC^+^) shows the typical characteristics of an ABCB1 allocrite and may be responsible for basal activity. Despite the low intrinsic p*K*_a_ value of the phosphate group in the pure phosphatidylcholine (PC) monolayers^[97]^, a small fraction of PC molecules may be protonated at the phosphate group in the overall negatively charged cytosolic membrane leaflet of cells. As the flipping rate of PC lipids (from the extracellular to intracellular membrane) is low^[98]^, and ABCB1 may cope approximately by flopping them back to the extracellular membrane leaflet, the concentration of POPC^+^ in the cytosolic membrane leaflet remains low and prevents inhibition of the transporter. ABCB1 may thus contribute to the maintenance of lipid asymmetry in biological membranes, as suggested earlier^[63,99]^. The particularly low basal activity in DPPC vesicles^[27]^ could be due to a very low concentration of the cationic species in the absence of negatively charged lipids, or if the zwitterionic form is also an allocrite, to the an excessive and thus inhibitory concentration of DPPC. Thus, basal ABCB1 activity may arise from flopping POPC^+^, or POPC in general.

### The factors influencing the steady-state ATP hydrolysis rate

#### Correlation between the steady-state ATP hydrolysis rate and allocrite affinity 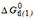


Stein and colleagues studied the correlation between kinetic parameters and the lipid-water partition coefficient as well as the van der Waals surface area of drugs in inside-out plasma membrane vesicles of CR1R12 cells^[96]^. With the exception of valinomycin, a good correlation between the surface area of drugs and the compound’s affinity to ABCB1 was observed. Similar data were provided by Sharom and colleagues^[99]^.

A good correlation between size-related parameters and the compound’s affinity to the transporter within the lipid phase 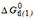 can be rationalized by assuming that increasing the surface area of the drug requires increasing the number of hydrogen-bonding groups to prevent aggregation. Therefore, increasing the molecular surface area (or molecular weight) roughly correlates with increasing the number of HBAs in ABCB1 (= O, -O-, -N <), which can interact with HBDs in the transporter. If one considers that valinomycin offers a number of π-electron donor groups for complex formation with potassium, it is no longer an outlier^[96]^. The correlation between HBAs and the molecular weight of allocrites is also displayed in Supplementary Figure 3. Thus, the observed decrease in activity with increasing affinity can be explained by weak dipolar interactions between the available HBAs in allocrites and the HBDs in the protein.

A reduced allocrite affinity with a concomitant increase in activity was also obtained by eliminating “anchor points” in the protein binding region, for example, by mutating amino acids able to form π-π stacking or hydrogen bonding interactions with allocrites. Compounds that were “inhibitors” in the native transporter became activators in the mutant^[100,101]^, supporting the inverse correlation between affinity 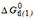 and the steady-state ATP hydrolysis rate. Thus, the steady-state ATP hydrolysis rate and transport decrease with an increasing number of weak dipolar interactions (see e.g., TMR and R6G in Figure 1 from Ref.^[102]^). Using a broader range of tetramethylrosamine (TMR) analogs and their xanthone precursors, Tombline and colleagues^[102]^ demonstrated that, in addition to the number of HBAs, the logP (octanol-water partition coefficient, used as a crude estimation of lipid-water partition coefficient) plays an additional role (e.g., Chart 2, compounds 14-16 in Ref.^[102]^). A strict correlation between the rate and 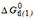 holds true only if 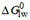 is rather constant. More diverse sets of compounds require the inclusion of 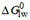 (see below).

#### The steady-state ATP hydrolysis rate (lnV_1_) decreases linearly with 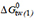


**Figure 5:**
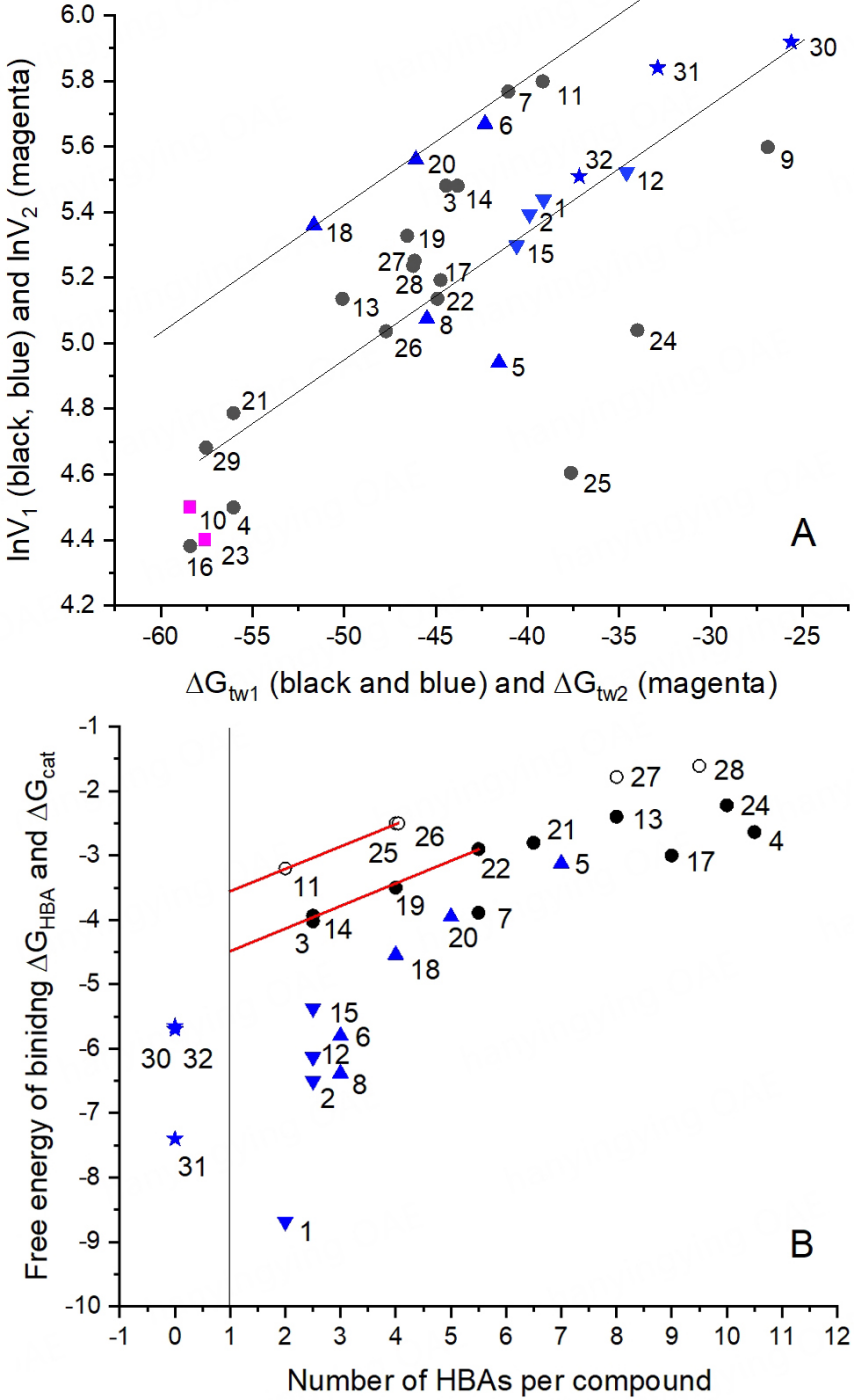
Correlations between maximum steady-state ATP hydrolysis rate and affinity in mouse embryo fibroblast membranes. (A): ln(*V*_1_) *vs.* the free energy of binding, 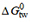. The maximum steady-state ATP hydrolysis rate, *V*_1_, is expressed as a percentage of the basal steady-state ATP hydrolysis rate taken as 100%. Data were obtained from phosphate release measurements: (1) amitriptyline, (2) chlorpromazine, (3) cis-flupenthixol, (4) cyclosporine A, (5) daunorubicin, (6) dibucaine, (7) diltiazem, (8) glivec, (9) lidocaine, (10) OC144-093, (11) progesterone, (12) promazine, (13) reserpine, (14) trifluoperazine, (15) trifluopromazine, (16) PSC 833, (17) vinblastine (1-17, from Ref.^[78]^), (18) amlodipine, (19) nimodipine, (20) verapamil (18-20, from Ref. ^[93]^), (21) sirolimus, (22) tacrolimus (21-22 from Simon Lang and A.S., unpublished results), (23) tariquidar (X. Li-Blatter and A.S., unpublished results), (24) etoposide (from Ref.^[66]^), (25) C_12_-maltoside, (26) C_13_-maltoside, (27) C_12_EO_8_, (28) Triton X-100, (29) Tween 80, (30) C_10_-TAC, (31) C_12_-TAC, (32) C_14_-TAC (25-32, from Ref.^[79]^). Black filled circles: neutral compounds or compounds exhibiting low charge (pK_a_ ≤ 8). Blue upward-pointing triangles: cationic compounds with intermediate charge (pK_a_ ≥ 8). Blue downward-pointing triangles: strongly charged cationic compounds (pK_a_ ≥ 9). (B): The free energy of binding per single cationic full charge, 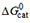, and single HBA, as a function of the number of patterns per compound 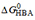. Compound numbers and symbols as in (A), black open circles, uncharged compounds. The free energy per hydrogen bond (y-axis) decreases with the increasing number of hydrogen bonds in patterns per compound (x-axis). We extrapolated to one hydrogen bond in the case of electrically neutral compounds and compounds with low charge (red lines).


An approximately linear decrease of the steady-state ATP hydrolysis rate, lnV_1_ (on a logarithmic scale), with decreasing free energy of binding 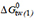 (or increasing allocrite affinity to the transporter) was observed with various data sets^[78,79,82]^. Figure 5A shows lnV_1 _*vs.* 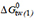 for very diverse types of allocrites, including moderately charged (on the upper diagonal line), highly charged (on the lower diagonal line), and essentially uncharged amphiphiles (between the two diagonal lines). Figure 5A, moreover, includes molecules of low amphiphilicity, such as PSC 833, cyclosporine A, OC144-093, and tariquidar, or molecules of unfavorable amphiphilicity, such as DDM^[79]^ (below the lower diagonal line). The strict linear dependence of lnV_1 _*vs.* 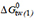 thus exists only within a specific charge group. To understand this phenomenon, we assessed the free energy contribution per single hydrogen bond, 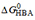, and per single charge, 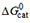.

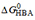
 was assessed by dividing 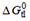 by the number of HBAs in type I and type II patterns of the different compounds (Figure 5B, y-axis). These values were plotted as a function of the number of HBAs per compound (Figure 5B, x-axis). A higher number of HBAs per compound was associated with a lower contribution per single HBA, suggesting that not all HBAs present contribute simultaneously to binding in the case of larger compounds with a higher number of HBAs. The value per single hydrogen bond was estimated by extrapolation [Figure 5B]. The values given in Figure 5B were evaluated for mouse embryo fibroblast membranes.

#### The free energy of binding per charge and HBA - comparison with literature data

To assess the affinity of a full cationic charge to ABCB1, we used the quaternary ammonium ion in C_m_-TACs. The free energy of binding per cationic charge to ABCB1 was assessed as 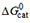 = -6.5 ± 0.7 kJ mol^-1^^[79]^ (see Figure 5B). The value is within the range of π-cation interactions^[103]^.

For electrically neutral detergents with a very peripheral location of the polar head group, and progesterone, which lacks a strong type I pattern, extrapolation to a single HBA yields 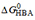 ≈ -3.5 kJ mol^-1 ^[Figure 5B]. For electrically neutral drugs likely penetrating more deeply into the membrane, extrapolation to a single hydrogen bond yields 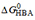 ≈ -4.5 kJ mol^-1^. Highly charged compounds such as amitriptyline (pK_a_ 9.4) exhibit more negative free energy of binding, 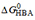 = -8.7 kJ mol^-1 ^[Figure 5B], owing to a charge contribution. Because most cationic drugs show lower pK_a_ values than amitriptyline, charge contributions are generally low and hydrogen bonding dominates.

The calculated free energy of hydrogen bond formation is on the order of -20 kJ/mol (see e.g.,^[104]^) and is thus much higher than the free energy per HBA assessed for ABCB1. However, our values agree well with the rare *measured* free energies of hydrogen bond formation in large membrane proteins. The H-bond strength of a single Cα-H···O H-bond in the transmembrane helical dimer of glycophorin A, located in the center of the membrane, was assessed as 3.7 kJ mol^-1^, using vibrational frequency shifts of dimeric and non-dimeric variants of glycophorin A, containing a deuterium-labeled Gly^[105]^. The average contribution of eight interhelical side-chain hydrogen-bonding interactions throughout bacteriorhodopsin, reconstituted in DMPC (1,2-dimyristoyl-sn-glycerol-3-phosphocholine)/CHAPSO {3[(3-cholamidopropyl) dimethylammonio]-2-hydroxy-1-propane-sulfonate}, was determined as 2.5 kJ mol^-1^^[106]^. The sign of free energy of binding depends on the reference state.

#### The role of amphiphilicity for the steady-state ATPase rate

Amphiphilicity is a qualitative term used to describe a molecule exhibiting a polar part and a non-polar, hydrophobic part. As shown previously, the steady-state ATP hydrolysis rate of detergents and drugs strongly depends on ratio *q* = 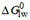/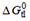 (ratio of the free energy of allocrite binding to the hydrophobic membrane and to the more polar transporter)^[79]^.

The ratio *q* can be considered as a quantitative description of a compound’s amphiphilicity. For ATPase-activating compounds such as the cationic C_m_-TACs, the ratio is *q* ≈ 3-4, for cationic drugs, *q* ≈ 3, and for electrically neutral detergents, *q* ≈ 2.7. Electrically neutral detergents are particularly sensitive to the ratio *q*. Slight deviations to higher values (e.g., C_14_-maltoside with *q* ≈ 3) lead to a lack of activation/inhibition because the affinity to the membrane is too high. Lower values lead to inhibition [e.g., C_12_-maltoside (DDM) with* q *= 2.5]. Inhibitory drugs including cyclosporine A show low ratios (*q* ≈ 1) (see Supplementary Information to Ref.^[79]^).

#### Conclusions on the mechanism of transport

Altogether, we demonstrated that the steady-state ATPase hydrolysis rate decreases with increasing affinity of the allocrite from water to the transporter 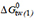. However, the rate is particularly sensitive to charge and amphiphilicity. Compounds with two HBAs and a very weak cationic charge or only two HBAs, providing a free energy of binding comparable to that of a single cationic point charge of 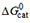 = -6.5 ± 0.7 kJ mol^-1^, induce a higher ATP hydrolysis rate than the latter. Weak dipolar interactions are thus more favorable for transport than a single strong cation-π interaction. Weak interactions thus may allow gliding of the polar part of the allocrite across the transporter binding region towards the center of the transporter in the middle of the membrane (which exhibits the lowest dielectric constant and thus the highest affinity to the transporter) and may facilitate flopping. While the polar part of allocrites forms weak electrostatic interactions with the transporter, the hydrophobic part seems to remain associated with the lipid membrane, as proposed earlier^[81]^. The slow-down in the steady-state ATP hydrolysis rate with reduced amphiphilicity is consistent with such an interfacial flopping process. An interfacial transport process has been described for the oligosaccharide transporter PglK with two ATPγS molecules bound^[107]^.

## ATP HYDROLYSIS IN RECONSTITUTED PROTEOLIPOSOMES AND DETERGENT MICELLES

### Proteoliposomes with residual detergents show higher *K_m_ (K_1_)* and lower *V_max_ (V_1_)* values

The concentrations of half-maximum activation *K*_m_ (Michaelis-Menten constant) or *K*_1_ [Supplementary Equation 12] of specific allocrites in proteoliposomes are often distinctly higher than in natural plasma membrane vesicles. If ABCB1 is reconstituted into lipid vesicles, detergents are used. Even though such detergents are usually removed after reconstitution, residual detergents may remain. Reconstitution of ABCB1 by solubilization in octyl glucoside (OG) in the presence of *Escherichia coli* lipids and subsequent titration with verapamil yielded an almost 10-fold higher concentration of maximum activation (e.g.,^[108,109]^). Still higher concentrations of maximum activation (e.g.,^[71]^) or even a loss of activity^[91]^ were observed when DDM was used. Callaghan and colleagues prevented “inactivation” by a careful reconstitution protocol that included excess crude lipid mixtures and extensive gel filtration to eliminate DDM^[91]^. The often-observed shift of *K*_m_ values to higher concentrations is caused by competitive inhibition of the allocrite (e.g., verapamil)^[82]^. An increase in *K*_m_, combined with a decrease in *V*_max_, is typical for competitive inhibition. The inhibitory power of detergents increases in the order CHAPS < OG < DDM^[27]^ (OG and DDM^[79]^, CHAPS: X.L. and A.S., unpublished results) [Figure 4].

The loss of activity of transporters reconstituted in lipid bilayers is thus caused by residual detergents such as OG and particularly DDM that act as competitive inhibitors. ATPase activity is regained by dilution or squeezing out (see, e.g., effects observed by addition of cholesterol in Ref.^[110,111]^).

### Detergent micelles show higher *K_m_ (K_1_)* and higher *V_max_ (V_1_)* values

The basal ATPase activity of Sav1866, reconstituted in liposomes, increased two- to threefold upon the addition of detergents above their critical micelle concentration (CMC), which led to the formation of mixed micelles (see Ref.^[32]^, Figure 5 therein).

Ambudkar and colleagues^[112]^ compared the basal ATPase activity of mP-gp (the murine analog of ABCB1) in native High Five insect cell membranes (42 to 54 nmol P_i_/min/mg of protein) and in DDM micelles (79 to 83 nmol P_i_/min/mg of protein). In DDM micelles, the basal ATPase activity was again about twice as high as in insect cell membranes. Moreover, a 30-150-fold decrease in the apparent affinity for verapamil and cyclic peptide inhibitor QZ59-SSS was observed in detergent micelles compared with that in native or artificial membranes. Consequently, the cyclic peptide “inhibitor” QZ59-SSS and the modulators zosuquidar, tariquidar, and elacridar (inhibitors in lipid vesicles with IC_50_ values in the 10-40 nM range) stimulated the ATPase activity of purified human or mouse P-gp in DDM micelles.

Micelles exhibit higher dielectric constants, ε_m_, than lipid vesicles, leading to a substantial decrease in allocrite affinity to the transporter (see Supplementary Equations 9-11) and a concomitant increase in ATPase activity and transport, as expected. Thus, ABCB1 remains functional in a micellar environment, although transport becomes Sisyphean because of the low lateral packing density, π_M_, of micelles.

### Cholesterol enhances the membrane packing density and allocrite affinity

The addition of cholesterol has often enhanced ABCB1 activity (see, e.g., effects observed by addition of cholesterol in Ref.^[110,111]^). The addition of lipids generally dilutes residual inhibitory detergents such as OG and DDM. Densely packed lipids, including cholesterol, may in addition squeeze detergents out of the lipid bilayer, which leads to enhanced ATPase activity.

However, the specific effect of cholesterol (in the absence of detergents) is to slightly reduce the steady-state ATP hydrolysis rate^[84,94]^. Cholesterol enhances the lateral membrane packing density, π_M_, and reduces the dielectric constant, ε_m_, of a lipid bilayer (see above), with the consequence of an enhanced dipolar attraction of the allocrite to the transporter within the membrane.

These predictions are consistent with experiments by Ueda and colleagues^[113]^, who measured the modulation of drug-stimulated ATPase activity of ABCB1 by cholesterol in the absence of OG or DDM. They reconstituted ABCB1 in membranes with different cholesterol contents (*C*_ch_ = 0%-20% w/w) and used ten allocrites with increasing molecular weights from 345 to 1111 Da (see Ref.^[113]^, Table 1 therein). For small molecules (molecular mass < 500 Da), *K*_m_ decreased (i.e., the affinity to the transporter increased) by about a factor of two with increasing cholesterol content. For larger molecules, *K*_m_ remained approximately constant, and for the largest molecule, *K*_m_ even increased slightly with increasing cholesterol content. For small compounds, lipid-water partitioning is not limiting and the affinity to the transporter within the membrane increases with decreasing dielectric constant ε_m_. Conversely, in the case of the largest compounds, the affinity to the transporter slightly decreased because partitioning into the membrane became the limiting factor. With increasing cholesterol content, the activity, *V*_max_, clearly decreased for large molecules with many HBAs, whereas it remained approximately constant for small molecules. Although the effects are minor, the data^[113]^ perfectly agree with the above expectations. Sharom and colleagues obtained related results and also demonstrated that “the cholesterol content of the membrane has only a modest influence on both the basal and the drug-stimulated ATPase activity of P-gp”^[114]^.

#### The consequences of using methyl-β-cyclodextrin (mβCD) for cholesterol elimination

Elimination of cholesterol by mβCD often leads to a strong decrease in ABCB1 activity. On the basis of this observation, it was concluded that cholesterol must be strongly ABCB1-enhancing, but this is not the case (see above). Elimination of cholesterol with mβCD is complex. The cyclic polysaccharide has a central hydrophobic cavity that can be occupied by cholesterol, lipids, or any other hydrophobic or amphiphilic molecule. In its empty form, mβCD engulfs cholesterol from the hydrophilic end, thereby partially penetrating into the head group region of the lipid membrane. Sharom and colleagues^[114]^ found a decrease in ABCB1 activity with increasing concentrations of mβCD in CH^R^B30 plasma membrane vesicles, in DMPC proteoliposomes, and in CHAPS micelles, independent of cholesterol and suggested the possibility of a direct interaction between ABCB1 and mβCD^[114]^. mβCD carries multiple hydrogen bond acceptor patterns, and therefore direct interaction with ABCB1 is highly likely. It seems to start already at low concentrations (X.L-B and A. S. unpublished results). Because membranes disintegrate at higher mβCD concentrations, obtaining definitive experimental proof of this is difficult.

To inhibit endogenous cholesterol synthesis, lovastatin was used in addition to mβCD^[115]^. Lovastatin is also a modulator of ABCB1 directly inhibiting ABCB1 at the concentrations used (A.S. unpublished results). Thus, the strong effects observed upon cholesterol elimination with mβCD are likely caused by direct inhibition of ABCB1 by mβCD at low concentrations and by general delipidation at higher concentrations.

Thus, cholesterol elimination with mβCD strongly reduces ATPase activity, but cholesterol supplementation in a biological membrane would not enhance it.

## ATP HYDROLYSIS IN LIVING CELLS

At this point, the altered “*membrane permeability*”^[1]^ of cells in the presence of ABCB1 becomes relevant. Whereas in inside-out vesicles, active transport and passive diffusion work in the same direction, they work in opposite directions in cells. This phenomenon was described as the “pump-leak effect”^[116]^. Here, the balance is in favor of export (pump) in the case of large molecules, and in favor of influx (leak) in the case of smaller ones^[58]^. The comparison of ATPase activity measurements in inside-out plasma membrane vesicles and cells is therefore of special interest.

Cells cultured in the presence of glucose generally work under glycolytic conditions^[117]^. Under these conditions, the extrusion of one lactate corresponds to one ATP synthesized. As ATP is synthesized according to requirements, ATP hydrolysis can be monitored by measuring the steady-state extracellular acidification rate (ECAR) using a Cytosensor microphysiometer^[117,118]^. The drug-stimulated ABCB1 activity was obtained by comparing MDR1-transfected mouse embryo fibroblasts (NIH-MDR1-G185 cells) with the corresponding wild-type cells (NIH-3T3 cells). If the energy requirement is enhanced, cells possess the ability to dynamically switch to oxidative phosphorylation (or respiration)^[119]^. To remain under glycolytic conditions and prevent potentially toxic side effects, drugs were applied for short time intervals of 2-3 min^[59,120]^ and were washed out after each stimulation (see Supplementary Figure 4).

### Small molecules: ABCB1 titration curves in cells and vesicles are similar

**Figure 6:**
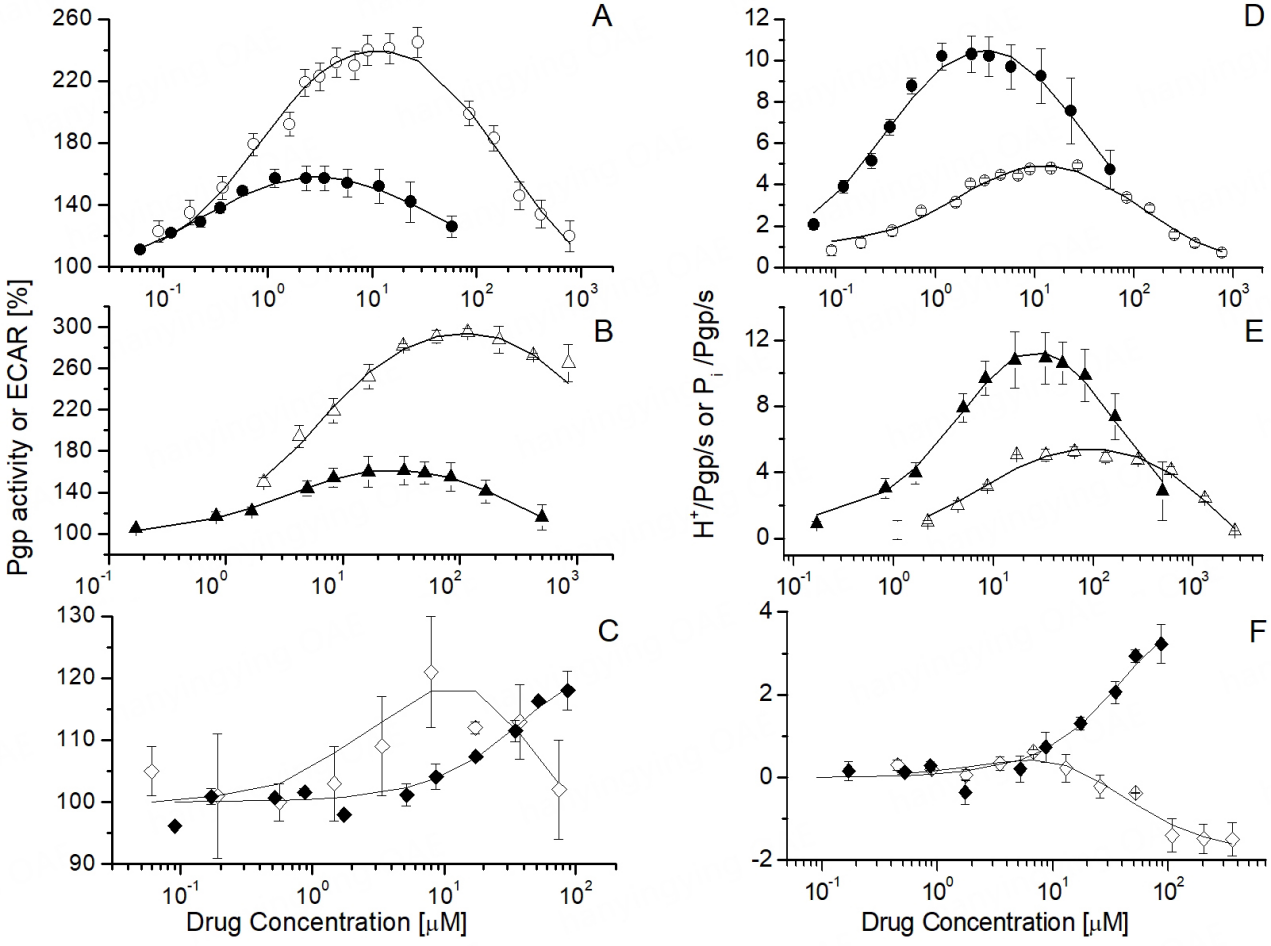
ABCB1 ATPase activity in NIH-MDR1-G185 cells (filled symbols) and inside-out plasma membrane vesicles (open symbols) induced by three drugs. (A-C) ATPase activity expressed as percent of the basal value in the absence of drugs. (D-F) ATPase activity expressed as protons (i.e., lactic acid) or phosphate released per ABCB1 per second. Calculations are based on the expression level of ABCB1 determined for NIH-MDR1-G185 cells ^[124]^. (A and D) Verapamil 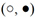; (B and E) diltiazem 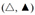, and (C and F) 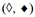 daunorubicin. Lines are fits to Supplementary Equation 12. Standard deviations are shown (taken from Ref.^[69]^). With copyright permission from BBA.

Small cationic drugs equilibrate rapidly between the inner and outer plasma membrane leaflets, and thus the concentrations of half-maximum activation *K*_1_ are similar in cells and inside-out vesicles [Figure 6A and D or B and E]. The slightly lower *K*_1_ values in cells are likely due to the somewhat lower cytosolic free magnesium ion concentration in cells and thus to a somewhat more negative membrane potential compared with that in vesicles^[32,112]^. Notably, the ATPase activity induced by small allocrites was more than twofold higher in cells than in plasma membrane vesicles of the same cells, even at the short stimulation times^[69]^. Significantly higher steady-state ATP hydrolysis rates in cells than in proteoliposomes [Figure 6C and F] were also measured for CFTR (ABCC7)^[14]^.

### Large molecules: titration curves in cells and vesicles differ, revealing transport

For slowly diffusing compounds, such as daunorubicin (MM: 527.5 Da), a known ABCB1 “substrate” [Figure 6B], the ATPase activity profiles of inside-out vesicles and cells differed distinctly. At identical aqueous concentrations (e.g., C_dau_ = 1 μM), daunorubicin inhibited ABCB1 activity in inside-out vesicles and activated it in living cells. The drug concentration in the cytosolic membrane leaflet of cells was estimated according to the new *K*_1_ value to be approximately one hundreds that in the cytosolic membrane leaflet of inside-out plasma membrane vesicles^[69]^. In the case of daunorubicin and other “substrates”, ABCB1 can maintain a concentration gradient (between the inner and outer plasma membrane leaflets) or, in other words, can cope with influx. Transport, reflected by a substantially reduced *K*_1_ value in the cytosolic membrane leaflet of cells, was also observed for Tween (reduced by about one-thousandth)^[69]^ and vinblastine (reduced by about one-tenth) (see Ref.^[78]^, Figure 3A-E therein). Interestingly, even detergents such as Triton X-100 and C_12_EO_8_ showed a reduction to approximately one-tenth in the cytosolic membrane leaflet of cells^[69]^.

ABCB1 efficiently competes with passive influx of large allocrites, that is “substrates” (see Ref.^[35]^). Therefore their concentration in the cytosolic plasma membrane leaflet of cells can be significantly lower than in the cytosolic leaflet of inside-out plasma membrane vesicles of the same cells*.* These measurements provide the first unequivocal insight into the correlation between ATPase activity and transport.

### Stoichiometry: one ATP hydrolyzed per allocrite transported

The stoichiometry of ATP-driven ion transporters moving ions from the aqueous phase at one side of the membrane to the aqueous phase at the other side of the membrane has long been determined^[121]^. Assessing the stoichiometry of ATP-driven allocrite transport by ABCB1 has proven significantly more challenging. Allocrites are captured in the cytosolic membrane leaflet, and are flopped to the extracellular leaflet^[122]^. From the extracellular leaflet, they eventually partition into the aqueous phase according to their lipid-water partition coefficient. Thus, many drugs accumulate within membranes.

To circumvent the problems caused by allocrite partitioning into the membrane and passive diffusion across the membrane, Eytan *et al.* measured the ATP-dependent uptake of the ^86^Rb^+^-valinomycine complex into proteoliposomes^[123]^. They suggested 1.2-2 ATPs hydrolyzed per transported ^86^Rb^+^-valinomycine complex.

Stein and colleagues^[124]^ used vinblastine, a known “substrate,” as a test molecule. Vinblastine exhibits a comparatively low lipid-water partition coefficient, *K*_lw_ ≈ 15 M^-1^ (in the absence of DMSO) [Table 1] (Li-Blatter X, unpublished results), a relatively large cross-sectional area (A_D_ = 140 Å^2^), and accordingly relatively slow diffusion. Maximum outward pumping from NIH-MDR1-G185 mouse embryo fibroblasts loaded with vinblastine was assessed as 2.1 × 10^6^ molecules s^-1^ cell^-1^ with a turnover of 1.1 molecules s^-1^. Vinblastine uptake in the absence and presence of the inhibitor verapamil (at inhibitory concentration, c = 50 μM) yielded maximum outward pumping of 2.73 × 10^6^ molecules s^-1^ cell^-1^. The turnover was 1.4 molecules s^-1^^[124]^. Comparing these values with the steady-state ATP hydrolysis rate in inside-out plasma membrane vesicles (*V*_max_ = 3.5 ABCB1 molecules^-1^ s^-1^) suggested that about two ATPs hydrolyzed per vinblastine transported^[124]^. At the low concentrations used for data evaluation (C_vin_ ≤ 10 μM) and the short stimulation times^[124]^, toxicity was most likely negligible.

**Table 1:** ABCB1 allocrites used as inhibitors

**Allocrites**	**MW**	**Log P^4)^**	***K*** **_lw_**	***K*** **_tl(2)_**	***K*** **_tw(2)_**	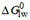	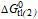	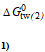
	[Da]		[M^-1^]	[M^-1^]	[M^-1^]	[kJmol^-1^]	[kJmol^-1^]	[kJmol^-1^]
Cyclosporine A^[48]^	1202.6	2.9	5.2*10^3^	1.3*10^2^	6.7*10^5^	-32.4	-12.5	-44.9
*Taxol^2)^*	*853.92*	~3						
*Vinblastin^2)^* ^[78]^	*811.0*	*3.7*	1.5*10^1^	1.6*10^3^	2.4*10^4^	*-17.3*	*-19.1*	*-36.4*
Encequidar^3)^	688.7	5.8	-	-	-	-	-	-
Tariquidar^3)[Li-Blatter X^^,^^ unpbl.]^	646.7	6.1	3.8*10^4^	2.4*10^3^	9.1*10^7^	-37.5	-20.1	-57.6
Elacridar^3)^	563.6	5.6	-	-	-	-	-	-
Zosuquidar^3)^	527.6	4.9	-	-	-	-	-	-
OC144-093^3)[78]^	494.7	7.3	-	-	1.1*10^6^	-	-	-46.1
Verapamil^[93]^	454.6	3.8	4.7*10^2^	5.810^1^	2.7*10^4^	-26.2	-10.4	-36.6

^1)^All allocrites listed inhibit as dimers, except cyclosporine A, which may inhibit as a monomer. The free energy of binding of the second, inhibitory molecule G_tw(2)_ is always less negative than the free energy of binding of the first, activating molecule ΔG_tw(1)_^[59]^. ^2)^Taxol and vinblastine are not useful as inhibitors, taxol because of its low solubility^[125]^ and vinblastine because of its relatively low affinity to the membrane (see Table 1). ^3)^The high affinity of encequidar, tariquidar, elacridar, zosuquidar, and OC144-093 is not due to a particularly high affinity to the transporter, but to a particularly high. ^4)^LogP values are from https://pubchem.ncbi.nlm.nih.gov

We monitored ATP hydrolysis in the same NIH-MDR-G185 cells and the corresponding wild-type cells as a function of vinblastine concentration using a Cytosensor microphysiometer^[78]^. The concentration of half-maximum activation of vinblastine, *K*_1_, in live cells shifted to an approximately 10-fold higher concentration than that in the inside-out plasma membrane vesicles of the same cells. Although the steady-state ATPase rate was higher in cells than in plasma membrane vesicles, the turnover number at C_vin_ ≤ 10 μM was only about 1.4 s^-1^ because of the *K*_1_ shift from 1.6 μM in inside-out vesicles to about 16 μM in cells. The kinetic data obtained in live cells support a one-to-one stoichiometry. Using rhodamine 123 as a “substrate,” Shapiro and Ling^[126]^ also proposed a one-to-one stoichiometry. Further arguments supporting a one-to-one stoichiometry are discussed elsewhere^[33]^. Under inhibitory conditions, where two allocrites are bound, the hydrolysis of one ATP most likely allows flopping of two allocrites, although at a low rate.

## ABCB1 INHIBITION

### A broad binding region allows for allosteric or competitive inhibition

In principle, any allocrite that can reach the inhibitory phase of a bell-shaped ATPase activity titration curve can act as an inhibitor (see, e.g., verapamil in Figure 4), provided it is soluble at the concentrations required. Inhibition is moreover obtained by compound combinations. Stein and colleagues^[127]^ assessed three categories of interactions in the drug binding region of ABCB1: (i) cooperative stimulation between verapamil and amphiphilic molecules smaller in size than verapamil (e.g., progesterone); (ii) allosteric inhibition between verapamil and molecules of similar size (e.g., daunorubicin); and (iii) competitive inhibition between verapamil and molecules larger in size such as cyclosporine A (see Supplementary Figure 3). Competitive inhibition was moreover observed between vinblastine, verapamil, cyclosporine A, and lipids^[99]^ or between verapamil, cyclosporine A, and the detergents Triton X-100, C_12_EO_8_, and Tween 80^[82]^. Depending on the concentration applied, detergents such as polyethylene glycol and Tween that are often used as excipients in drug formulations (see, e.g.,^[128]^, Figure 5) can also act as inhibitors of ABCB1. The possibility of accommodating a range of compounds in different combinations reveals *broad binding regions*^[127]^ with multiple anchor points (i.e., HBDs and unsaturated rings). Large ABCB1 binding regions were also observed by cryo-EM (e.g.,^[71]^).

If two identical molecules bind, the second inhibitory molecule has a lower affinity to the transporter than the activating first molecule^[59]^. Under most circumstances, “inhibition” is therefore a transient slowing-down of the transporter that rapidly fades away by dilution (see Supplementary Figure 4). This is in contrast to inhibitors of receptors (that work according to the lock-key principle). They generally show higher affinities to the receptor than the activators (see, e.g., the neurokinin-1 receptor. It binds its activator, substance P, an amphiphilic pain transmitter peptide, in the nanomolar concentration range^[129]^ and inhibitors in the sub-nanomolar concentration range^[130]^).

### The characteristic features of ABCB1 inhibitors

The principle feature of inhibitors is a very negative 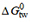 value. This is achieved with compounds exhibiting either a particularly high affinity to the transporter within the membrane, 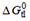 (due to numerous HBAs such as cyclosporine A), or a very negative 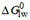 value (high logP or logD values) and an intermediate affinity to the transporter, 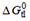 (e.g., encequidar, tariquidar, elacridar, zosuquidar, and OC144-093) [Table 1]. The sheer length and partial rigidity of some of the newer inhibitors may additionally impede rapid flopping.

A further feature inducing ABCB1 inhibition is the above-discussed inappropriate (e.g., DDM) or low amphiphilicity, which is quite common among inhibitors (e.g., cyclosporine A). A large list of inhibitors is given in a review by Artursson and colleagues^[131]^. In addition to many hydrophobic examples, dipyramidole, which is a relatively hydrophilic and non-amphiphilic compound, is listed as an ABCB1 inhibitor^[131]^.

#### “Transport substrate or inhibitor”?

Alam and colleagues observed that zosuquidar (“inhibitor”) and taxol (“substrate”) bind to the same pocket and asked about “how ABCB1 distinguishes transport substrates from inhibitors and how these compounds exert opposite effects on the ATPase activity”^[72]^. These questions can be answered using the present data [Table 1]. Zosuquidar is very hydrophobic (high LogP) and has a rather small cross-sectional area perpendicular to the axis of amphiphilicity^[132]^. Thus it likely exhibits a very negative 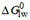. Owing to the high membrane concentration, two molecules of zosuquidar will bind to the transporter already at low aqueous concentrations. In comparison, taxol and vinblastine exhibit rather large cross-sectional areas. Moreover, they are relatively hydrophilic and exhibit a much less negative 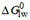. Thus, they will barely reach the concentration of half-maximum inhibition, *K*_2_. The latter molecules also show a low flux across the membrane and are therefore prone to be exported by ABCB1 in cells (for vinblastine^[78]^ and taxol^[133]^), which further enhances *K*_2_.

Understanding ABCB1 inhibition is fundamental for understanding ABCB1 function. ABCB1 inhibition plays a significant and possibly underestimated role in drug-drug interactions resulting from polypharmacy^[35]^. Note, that systemic ABCB1 inhibition to enhance cancer drug absorption was not successful in clinical trials^[134,135]^.

## UNIDIRECTIONAL TRANSPORT CYCLE FOR ABCB1 ALLOCRITES

**Figure 7:**
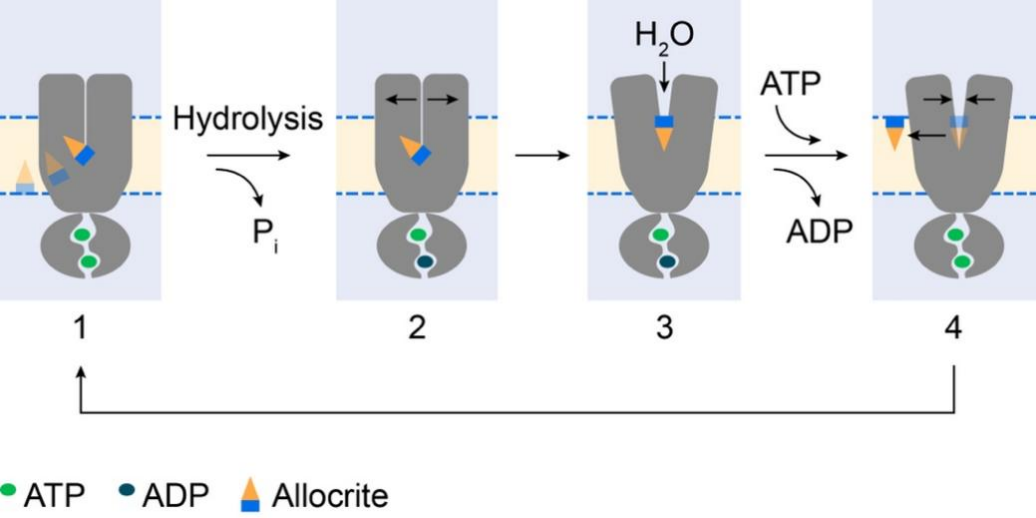
Unidirectional transport cycle in ABC exporters is driven by weak dipolar interactions between the allocrite and the transporter. (1) With two ATPs bound to the NBDs, the TMDs are in the outward closed resting state. The amphiphilic allocrite with HBAs in the polar part (blue) is attracted to the transporter via weak dipolar interactions. The polar groups are then drawn towards the center of the membrane (ε_m_ ≈ 2). (2) Hydrolysis of ATP and release of inorganic phosphate (P_i_) lead to an asymmetric occupation state of the NBDs, which initiates the opening of the TMDs. (3) The TMDs adopt an outward open conformation, which allows influx of water molecules. The allocrite flops (i.e., it turns around to expose its polar part towards the aqueous phase). The hydrogen bonds between the allocrite and the transporter are dissolved. The binding of ATP to the empty NBS restores the symmetric occupancy state and favors the outward closed conformation. The allocrites on the extracellular side of the cavity between the TMDs are squeezed out to the membrane (see also Ref.^[33]^).

Combining the physicochemical insights gained in this review with our previous molecular dynamics simulations^[33]^ yields the transport cycle for ABCB1 schematically summarized in Figure 7: (i) Allocrites partition into the lipid membrane, and accumulate in the cytosolic membrane leaflet with the polar part located in the interfacial membrane region. The polar part of the allocrite, carrying at least one type 1 pattern (with HBAs, see Figure 1), is attracted by the transporter and likely glides along the numerous HBDs at the protein surface towards the core of the membrane, where the attraction is highest, due to the lowest dielectric constant. (ii) Allocrite binding likely elicits a strain on the transporter that leads to ATP hydrolysis and opening of the transporter towards the extracellular side. (iii) Inflowing water molecules compete for dipolar interactions with the transporter and the allocrite and induce de-binding of the allocrite, a process described as the solvation exchange mechanism by Omote and Al-Shawi^[136]^. As water fills the newly formed cavity, allocrites orient with their polar groups towards the aqueous phase (i.e., they flop). (iv) Upon ATP rebinding, the transporter closes extracellularly. In this way, the potentially remaining allocrite is squeezed out of the transporter into the outer membrane leaflet. It then either diffuses into the aqueous phase or restarts the flip-flop cycle until it is degraded by other enzymes such as CYP3A4^[137]^, attracting allocrites within the membrane by the same weak dipolar interactions. A more detailed description of the “*unidirectional ABCB1 transport cycle driven by weak dipolar interactions*,” including supporting experimental data, is given in the Supplementary Materials. The rate-limiting step for the overall ATPase activity was previously suggested to be either the de-binding of the ligand or a conformational change of the enzyme, impeded by a bulky substrate^[96]^. We observed that the rates of ATPase activity and transport are lower for compounds with a higher affinity to the transporter, for more than one compound bound to the transporter, and for compounds of low amphiphilicity that are not easily oriented at a protein-water interface. Our findings thus suggest that the de-binding and flopping process is rate-limiting, supporting the early suggestions. A more detailed description, including experimental results, is given in the Supplementary Materials.

## CONCLUSIONS

*Thermodynamics:* The *amphiphilicity* of allocrites^[1] ^implies their accumulation in membranes. In the 1990s, ABCB1 was demonstrated to indeed attract its allocrites within the membrane. An allocrite recognition mechanism based on weak dipolar interactions that works in membranes was proposed. It explains the polyspecificity of ABCB1. With the advent of the first ABCB1 structures, the focus shifted to the protein *only*. In this context, the transporter was often treated conceptually as a receptor, binding its allocrites from the aqueous phase. With this review, we demonstrate that* understanding the nature of allocrite-ABCB1 interactions requires inclusion of the transporter environment*. The binding process starts with partitioning of the amphiphilic allocrite from the aqueous phase into the lipid membrane (step I). Partitioning is driven by classical hydrophobic interactions in the case of electrically neutral amphiphiles and by non-classical hydrophobic interactions in the case of cationic amphiphiles. Once in the membrane, the polar part of the allocrite (with HBAs) is attracted to the transporter (with HBDs and π-electron systems) (step II). Step I and Step II were quantified in terms of the free energies of binding 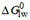 and 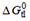. The sum of these two free energies yields the overall free energy of allocrite binding from water to the transporter, 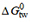. Whereas partitioning (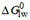) *decreases* with increasing lateral packing density of the membrane, weak electrostatic attraction to the transporter (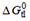) was shown to *increase* with increasing lateral packing density and concomitantly decreasing dielectric constant of the membrane. Owing to these compensatory mechanisms regarding the membrane packing density, the concentration of half-maximum activation, *K*_1_, changes only moderately in biological membranes.

*Kinetics*: P-glycoprotein was shown to alter the *membrane permeability*^[1]^, which suggests the presence of two competing processes, passive diffusion into the cell and active transport out of the cell. Theoretically, these two competing processes can be described rather easily in different ways: Ref.^[116]^ or Ref.^[58]^. Demonstrating these phenomena in a single experiment was more difficult and required ATPase activity measurements with large slowly diffusing allocrites in ABCB1-overexpressing cells as well as large slowly diffusing allocrites. These measurements revealed a significant concentration decrease in the cytosolic membrane leaflet (reflected by higher *K*_1_ values than observed in inside-out plasma membrane vesicles of the same cells), which can be attributed to the flopping activity of ABCB1^[69]^. Combining the early drug efflux measurements^[124]^ with ATPase activity measurements in the same cells, we obtained a stoichiometry of one ATP hydrolyzed per allocrite transported. Moreover, we highlight the importance of bell-shaped ATPase activity curves that account for binding of a second allocrite to the transporter^[96,127]^. The concept of two allocrites binding to ABCB1 is indispensable for understanding ABCB1 inhibition as well as drug-drug interactions.

*Combining thermodynamics and kinetics yields insights into the nature of allocrite-transporter interactions: *Generally, the steady-state ATP hydrolysis rate decreases with increasing allocrite affinity 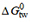 to the transporter [Figure 5A]. However, a detailed inspection of Figure 5A shows that allocrites with identical free energies of binding 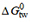 do not necessarily exhibit identical ATP hydrolysis rates. An allocrite undergoing weak, delocalized interactions with the transporter (e.g., via two HBAs and a weak charge) shows a *higher *steady-state ATP hydrolysis rate than a compound with a single strong point charge. Conversely, allocrites with inappropriate (e.g., DDM) or low amphiphilicity (e.g., cyclosporin A^[79]^ and some of the newer inhibitors such as elacridar) show *lower *steady-state ATP hydrolysis rates than expected from their 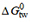 value. A substantial *increase* in the steady-state ATP hydrolysis rate is observed if ABCB1 is reconstituted in detergent micelles. Owing to the higher curvature and lower packing density, micelles exhibit higher dielectric constants than bilayers in lipid vesicles, which in turn significantly reduce dipolar interactions. Micelles, moreover, exhibit a lower “reservoir capacity for amphiphiles” (i.e., a negligible “
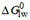
”). Therefore, allocrites that appear as inhibitors in membranes appear as activators in micelles. ABCB1 is thus a robust transporter (or rather a floppase) that perfectly adapts to membranes or micelles exhibiting very different lateral packing densities. The increase in *net permeability* in loosely packed systems is thus not due to a deficient transporter, but to the strongly enhanced passive diffusion. The clear dependence of the steady-state ATP hydrolysis rate on the nature and number of dipolar interactions, as well as on the dielectric constant of the lipid or micellar environment, points to a flopping process that, at least initially, takes place at *the interface between the lipid membrane and the protein*. The effects observed do not seem possible either at a protein-water interface (as suggested in alternating access models) or inside a protein channel, which is completely shielded from the lipid membrane.

*Combining the physical chemical insights gained in this review with our previous molecular dynamics simulations* ^[33]^ suggests that the ABCB1 transport cycle, including allocrite recognition, binding, and transport, is driven by weak dipolar interactions. Allocrite binding induces the hydrolysis of one ATP molecule, which leads to transporter opening towards the extracellular side. Influx of water molecules allows for allocrite flopping. ATP rebinding re-closes the transporter at the extracellular side and expels the potentially remaining allocrites. The individual steps can be quantified when taking into account that the transporter was optimized to operate in a membrane.

## DECLARATIONS

### Copyright

© The Author(s) 2023.
