# Supplementary material for: P-glycoprotein (ABCB1) - weak dipolar interactions provide the key to understanding allocrite recognition, binding, and transport
Source: Cancer Drug Resist. 2023 Jan 1;6(1):1–29. doi: 10.20517/cdr.2022.59 (PMC10105259; doi:10.20517/cdr.2022.59)
Supplement: Supplementary file 1 [file cdr-6-1-1-SupplementaryMaterials.pdf]

Seelig *et al*. *Cancer Drug Resist* 2023;6:1-29
DOI: 10.20517/cdr.2022.59

Supplementary Materials

BINDING CONSTANTS AND FREE ENERGIES OF BINDING

Binding of an allocrite from water to the transporter is defined by the transporter-water binding constant, K_{tw}, which can be expressed as the product of the lipid-water partition coefficient, K_{lw}, and the transporter-lipid binding constant, K_{tl} of the allocrite [Supplementary equation 1]. The corresponding free energies of binding, are given in [Supplementary equations 2-5]. R is the molar gas constant, T, the absolute temperature, and C_w, the molar concentration of water. For details see ref.[1].

$$K_{tw} = K_{lw} \cdot K_{tl} \quad \text{(S1)}$$

$$\Delta G^0_{tw} = \Delta G^0_{lw} + \Delta G^0_{tl} \quad \text{(S2)}$$

$$\Delta G^0_{tw} = -RT\,ln(C_w\,K_{tw}) \quad \text{(S3)}$$

$$\Delta G^0_{lw} = -RT\,ln(C_w\,K_{lw}) \quad \text{(S4)}$$

$$\Delta G^0_{tl} = -RT\,ln\,K_{tl} \quad \text{(S5)}$$

The lipid-water partition coefficient K_{lw} depends on the packing density π_m and the surface potential ψ_m of the membrane

The lipid-water partition coefficient K_{lw} can be assessed from surface activity measurements that provide the air-water partition coefficient, K_{aw} and the cross-sectional area of the compound, perpendicular to its amphiphilicity axis, A_D. With these parameters and the appropriate lateral membrane packing density π_m, the lipid-water partition coefficient can be calculated according to Supplementary equation 6, where N_A is the Avogadro number, R the molar gas constant and T the absolute temperature. Increasing the lateral membrane packing density of the membrane reduces

© The Author(s) 2023. Open Access This article is licensed under a Creative Commons Attribution 4.0 International License (https://creativecommons.org/licenses/by/4.0/), which permits unrestricted use, sharing, adaptation, distribution and reproduction in any medium or format, for any purpose, even commercially, as long as you give appropriate credit to the original author(s) and the source, provide a link to the Creative Commons license, and indicate if changes were made.

the lipid-water partition coefficient. K_{lw} increases with increasing negative surface potential ψ_m of the membrane [Supplementary equation 7], where F is the Faraday constant and z the charge of the allocrite[1] (Experimental details are given in refs.[2-4]). The corresponding free energy is given in Supplementary equation 8.

$$K_{lw} = K_{aw} \cdot e^{-\pi_m A_D N_A / RT} \quad (S6)$$

$$K_{lw} = K_{aw} \cdot e^{-\pi_m A_D N_A / RT} \cdot e^{-\psi_m Fz/RT} \quad (S7)$$

$$\Delta G^0_{lw} = \Delta G^0_{aw} - \pi_m A_D N_A - \psi_m Fz \quad (S8)$$

The transporter-lipid binding constant K_{tl} depends on the dielectric constant ε_m of the membrane

According to Coulomb's law the electrostatic potential, U, acting between two charges, q_D and q_A of opposite sign (where D stands for electron donors (or HBAs) and A for electron acceptors (or HBDs)), depends on the dielectric constant of the medium (i.e., membrane) ε_m , and the distance between the charges r, where ε_0 represents the permittivity of the vacuum [Supplementary equation 9].

$$U = q_D \cdot q_A / 4\pi\varepsilon_0 r \varepsilon_m \quad (S9)$$

At least two H-bonds (i.e., one pattern) are (is) required for allocrite binding to ABCB1. Binding occurs according to a modular principle, the total free energy of binding thus corresponds to the sum of all the individual free energies per hydrogen bond $\Delta G^0_{H_i}$ [Supplementary equation 10]. The corresponding binding constant is given below [Supplementary equation 11].

$$\Delta G^0_{tl} = -U_{total} = \sum_{i=1}^{n} \Delta G^0_{H_i} \quad (S10)$$

$$K_{tl} = K_0 \cdot e^{-U_{total}/RT} \quad (S11)$$

KINETIC TWO-SITE BINDING MODEL

To evaluate the bell-shaped binding constants, the model by Stein and coworkers[5] was used [equation 12]. It is based on the principles of Michaelis - Menten kinetics and takes into account a basal activity of ATP hydrolysis, V_0, in the absence of an exogenous allocrite,

$$V_S = \frac{K_1 K_2 V_0 + K_2 V_1 C_S + V_2 C_S^2}{K_1 K_2 + K_2 C_S + C_S^2} \qquad \text{(S12)}$$

where V_S is the ATPase activity as a function of the substrate (allocrite) concentration S, V_1, is the maximum velocity, if only activation occurred, and K_1 is the allocrite concentration that gives half this maximal increment in ATPase activity. V_2 is the activity at infinite concentration of the allocrite and K_2 is the substrate concentration which gives half-maximal reduction of ATPase activity from the value V_1.

EXPERIMENTAL ASSESSMENT OF BINDING CONSTANTS AND FREE ENERGIES OF BINDING FOR THE MEMBRANES USED

We measured the ABCB1 ATPase activity in live mouse embryo fibroblasts, NIH3T3 and NIH-MDR1-G185 cells (NIH3T3 cells transfected with the human *MDR1* gene[6]) (see e.g.[1], inside-out plasma membrane vesicles of the same cells e.g.,[7] or in both e.g.,[8]. The lateral membrane packing density of mouse embryo fibroblast membranes was determined as π_m = 30 mN/m at 37 °C[1]. The relatively low membrane packing density of mouse embryo fibroblast plasma membranes is consistent with the low cholesterol content of embryonic cell membranes[9].

The binding constants and the corresponding free energies of binding were assessed according to Supplementary equations 1-5. The lipid-water partition coefficients were assessed either by isothermal titration calorimetry or by surface activity measurements. If the lipid-water partition coefficient, K_{lw} was determined by isothermal titration calorimetry (ITC) (according to ref.[10]), POPC vesicles with a lateral packing density, π_m = 30 mN/m at 37°C[11], corresponding to the lateral packing density of mouse embryo fibroblast membranes at the same temperature were used as model systems. At room temperature POPC vesicles exhibit a packing density of π_m = 32 mN/m[12].

If the lipid-water partition coefficient, K_{lw} was derived from surface activity measurements (see refs.[2,3] according to Supplementary equations 6-8, the air-water partition coefficient, K_{aw}, the cross-sectional area, A_D, of the allocrite (both derived from surface activity measurements), and the independently determined lateral packing density, π_m of the membrane of interest (30 mN/m in the case of mouse embryo fibroblasts), were used.

We derived the transporter-water binding constant, $K_{tw(1)}$ from steady-state ATPase activity measurements according to equation 12, assuming that the reciprocal of the concentration of half-maximum activation K_1 is directly proportional to the binding constant of the substrate from water to the transporter, $K_{tw(1)}$ i.e., $1/K_1 = K_{tw(1)}$. An analogous equation can be derived for K_2[1].

The ATPase activity in mouse embryo fibroblast plasma membranes vesicles was measured according to Litman[13] (for details see[7]). The total protein concentration in these assays was 0.49 µM and the ABCB1 concentration was 5.4 nM.

The transporter-lipid binding constant K_{tl} was assessed as the quotient of the binding constants K_{tw} and K_{lw} [Supplementary equation 1].

Membrane concentration of allocrites at K_1 and K_2

For hydrophobic substrates, the membrane concentration of allocrites, C_m is usually much higher than the aqueous concentration, and is given by Supplementary equation 13, where C_m in [mole allocrite/mole lipid] ≈ [mole allocrite / liter lipid]. Replacing the aqueous substrate concentration C_w, in the simple Michaelis-Menten equation (see e.g. equation 9 in ref.[4]), by the membrane concentration C_m, (or mole fraction r_b) yields Supplementary equation 13 and adapted for a two-state model Supplementary equations 14 and 15. The latter equations allow estimating the molar concentration of allocrites at the concentrations of half-maximum ABCB1 activation and inhibition.

$$C_m \approx r_b = K_M \cdot K_{lw} \quad \text{(S13)}$$

$$r_b \approx K_1 \cdot K_{lw} \quad \text{(S14)}$$

$r_b \approx K_2 \cdot K_{lw}$ (S15)

BELL-SHAPED ATPase ACTIVITY CURVES

Supplementary Table 1. Change of membrane order parameter, S_{mol} upon addition of allocrites

The relevant allocrite concentration range was determined in terms of the mole fraction, $r_{b(1)}$ (or $r_{(2)}$) which is the product of the lipid-water partition coefficient, K_{lw}, and the concentration of half-maximum activation or inhibition K_1 (or K_2) [Supplementary equations 13-15].

Allocrite	Lipid NMR	D-label NMR	T (°C)	K_{lw} (M^{-1})	K_1 (M)	K_2 (M)	r_{b1}	r_{b2}	S_{mol} %at K_2*	Ref.
C6-gluc	DPPC	C2	45	7.2	$2.17\cdot10^{-2}$	$3.64\cdot10^{-2}$	$1.6\cdot10^{-1}$	$2.6\cdot10^{-1}$	-5	[11]
C10-gluc	DPPC	C2	45	$1.6\cdot10^{3}$	-	$3.26\cdot10^{-4}$	-	$5.2\cdot10^{-1}$	-5	[11]
C12-mal	POPC	C2	35	$5.0\cdot10^{3}$	-	$2.53\cdot10^{-5}$	-	$1.3\cdot10^{-1}$	-6	[11]
C12EO8	POPC	C9,C19	22	$4.8\cdot10^{3}$	$9.04\cdot10^{-7}$	$1.49\cdot10^{-5}$	$4.3\cdot10^{-3}$	$7.1\cdot10^{-2}$	-3	[14]
Verap.	POPC	C9,C10	22	$4.7\cdot10^{2}$	$9.50\cdot10^{-7}$	$3.70\cdot10^{-5}$	$4.5\cdot10^{-4}$	$1.7\cdot10^{-2}$	-17	[15]

*Decrease in the order parameter, taking the S_{mol} value in the absence of detergents or drugs as 100%.

ABCB1 ACTIVITY IN LIVE CELLS

The ATP consumed in a cell is immediately re-synthesized which allows monitoring the ATP hydrolysis rate by measuring the ATP synthesis rate. Most cells in culture are glycolytic (producing two molecules of ATP and two molecules of lactic acid per molecule of glucose consumed). Lactic acid diffuses out of the cell in the protonated form and gives rise to an extracellular acidification rate. This allows measuring ATP hydrolysis by a flow through Cytosensor microphysiometer[16]. The short stimulation times (2-3 min), followed by a drug flush-out period, before applying the next higher drug concentration allows keeping cells under glycolytic conditions and minimizes potential cytotoxic side effects. The same batch of cells could be used for several ABCB1 titrations, and yielded highly reproducible data[1]. For each concentration, the *second* stimulation point (at 260 s) was plotted for curve fitting (for detailed stimulation schedules see ref.[1], Figure 1 therein). Supplementary Figure 4 shows an example, for details see Figure legend. In the case of longer stimulation times or transporters with

higher activity than ABCB1 such as CFTR a Bionas microphysiometer was used that monitors the ECAR and OCR[17].

MOLECULAR DYNAMICS (MD) SIMULATIONS

We performed MD simulations with the Type IV fold protein Sav1866[18,19] which was used as a model system for ABCB1. In these simulations we observed the spontaneous closure of the TMD cavity observed in the nucleotide free apo-form of ABCB1. This suggests that the Sav conformation observed in the X-ray structure might correspond to a transition state. “Key interactions between helices 3 and 4 that are shown in our simulations to stabilize the outward-closed state are present in the X-ray structures, which nevertheless show the TMD in an outward-open state. In the crystal of Sav1866, the TMDs from neighboring proteins form contacts that could artificially stabilize the outward-open conformation (see ref.[20], Supplementary Figure 9 therein)”. “Variation of crystal contacts under different crystallization conditions have been suggested to explain the wide distribution of conformations observed in ABC transporter. Our simulations further suggest that lipids diffusing into the TMD cavity could stabilize the outward-open conformation (see ref.[20] and references therein)”

COMBINING MD SIMULATIONS WITH EXPERIMENTAL RESULTS

Resting state. With two ATPs bound, Sav1866 was outward closed[21].

The high intracellular ATP concentration (c_{ATP} = 1-10 mM)[22,23] and the comparatively low K_m values for ATP binding (c_{ATP} = 0.4 - 0.8 mM)[24,25] are consistent with two ATP molecules bound under resting conditions. Using fluorescent nucleotide derivatives Sharom and colleagues showed that the resting state P- glycoprotein bound two molecules of 2'(3')-O-(2,4,6-trinitrophenyl) adenosine 5'-triphosphate (TNP-ATP). An outward closed conformation was also observed for the two bacterial type IV ABC transporters McjD[26] and LmrA[27] (for review see also[28,29]).

ATP hydrolysis. Upon release of the inorganic phosphate, an asymmetric occupation state of the NBDs occurred, which initiated the opening of the TMDs towards the extracellular side[21].

According to Sharom and colleagues allocrite binding to the transporter induces ATP hydrolysis [24]. Using fluorescent nucleotide derivatives, they demonstrated that the vanadate-trapped transition state bound only one nucleotide molecule[30].

Post hydrolysis state. In the post hydrolysis state (i.e., asymmetric occupation state of the NBDs), the TMDs adopt an outward-open conformation that allows for water influx[21].

A modest outward opening of the TMDs was observed in the post hydrolysis state of LmrA in DDM micelles[31]. We assume that influx of water molecules, reduces allocrite-transporter interactions according to a solvation exchange mechanism and promotes allocrite flopping[32].

Resetting to the resting state. MD simulations showed that the binding of ATP to the empty NBS restores the symmetric occupancy state and favors the outward-closed conformation. The polar part of the allocrites on the extracellular side of the cavity between the TMDs is entirely squeezed out from the protein into the membrane.

Experimentally, a squeeze out into the extracellular membrane leaflet (not into the aqueous phase) was demonstrate e.g. by Omote and Al-Shawi Field[33]

Thus, in the simulations, the TMD cavity's spontaneous closure remains closed in the case of symmetric nucleotide occupancy states and reopens in the case of asymmetric occupancy states[21].

SUPPLEMENTARY FIGURES

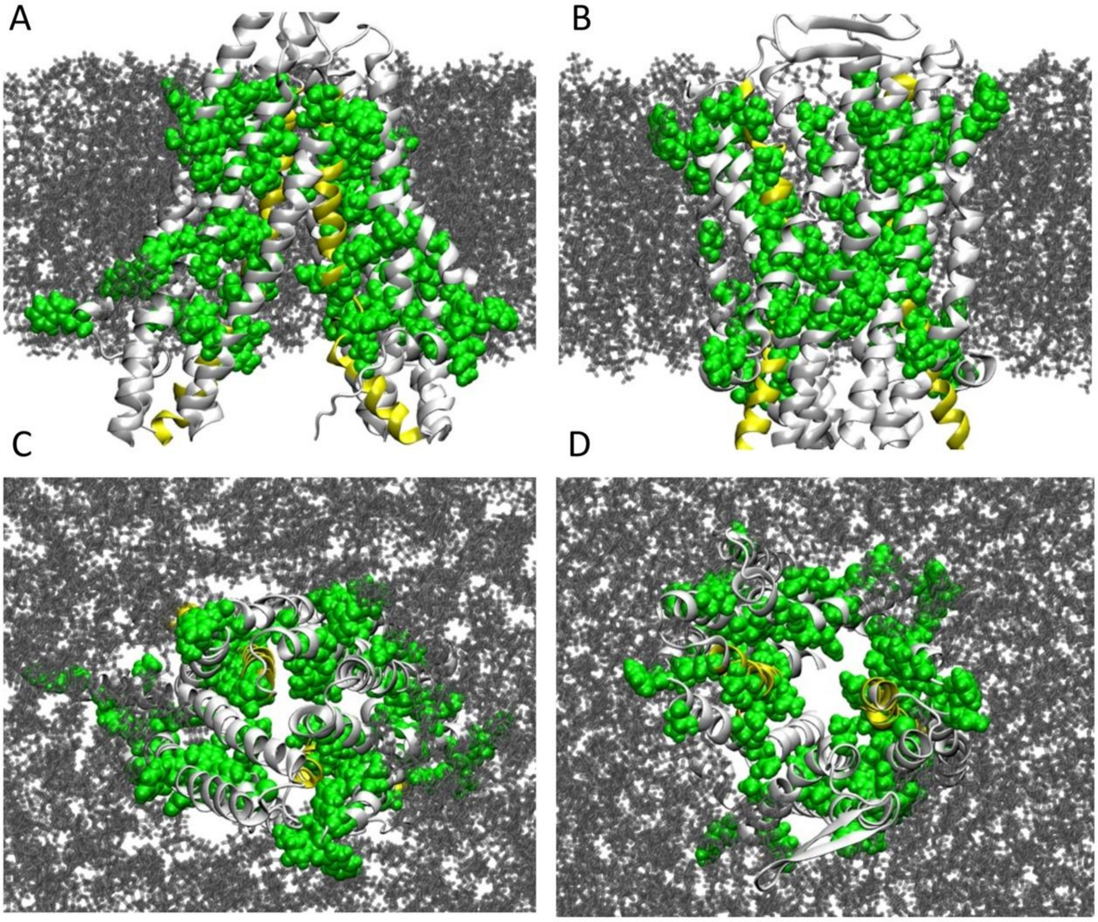

Supplementary Figure 1A-D. Amino acid residues with HBD side chains of ABCB1 at the level of the membrane (NBDs are truncated). Same structures as in Figure 2A-D. Hydrogen bonding amino acids (green) are shown with van der Waals radii. Lipids (black) are surrounding the transporter. The structures were modelled by Yanyan Xu in the context of ref.[34]

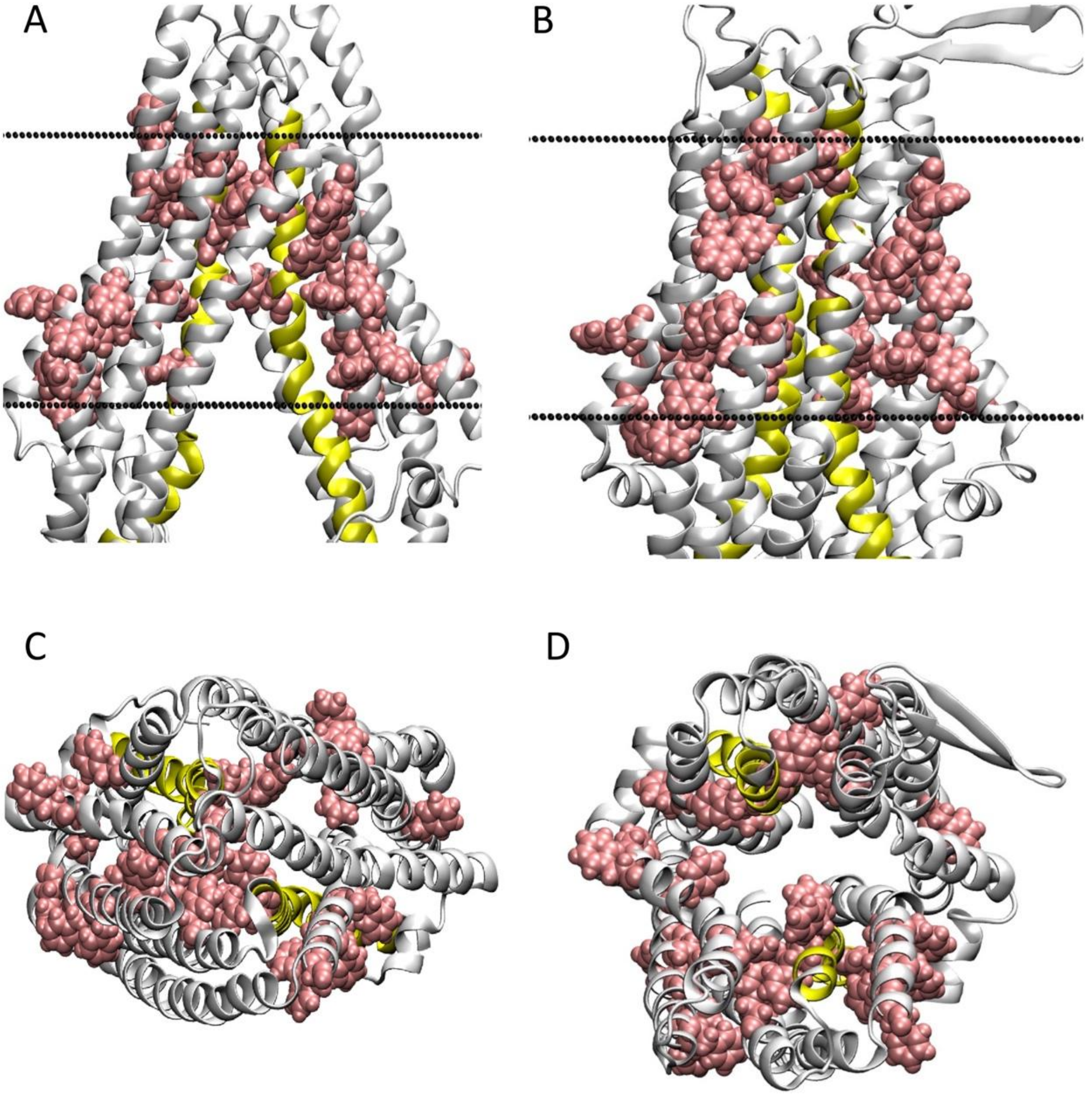

Supplementary Figure 2A-D. Phenyl residues of ABCB1 at the level of the membrane. Same structures as in Figure 2A-D. Phenyl residues (pink) are shown with van der Waals radii. The structures were modelled by Yanyan Xu in the context of ref.[34].

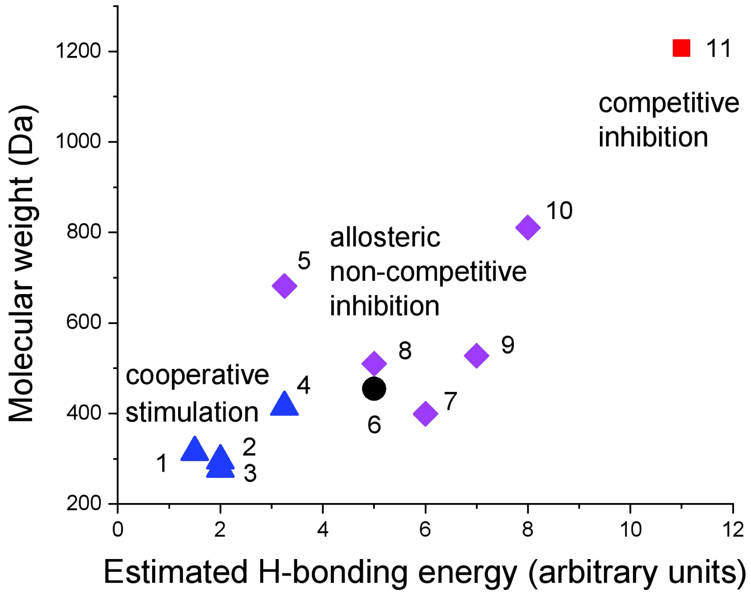

Supplementary Figure 3. Stimulatory or inhibitory interactions between verapamil and other compounds in correlation to their molecular weight and estimated hydrogen bonding energy (see[35]). Verapamil shows cooperative stimulation with compounds (1-4) (blue), allosteric, non-competitive inhibition with compounds (5, 7-10) (violet), and competitive inhibition with compound (11), (red). (1) progesterone, (2) propranolol, (3) amitryptiline, (4) diltiazem, (5) amiodarone (with two iodines), (6) verapamil, (7) colchicine, (8) gramicidin S, (9) daunorubicin, (10) vinblastine, (11) cyclosporine A. Experimental data are taken from Litman *et. al.*[36].

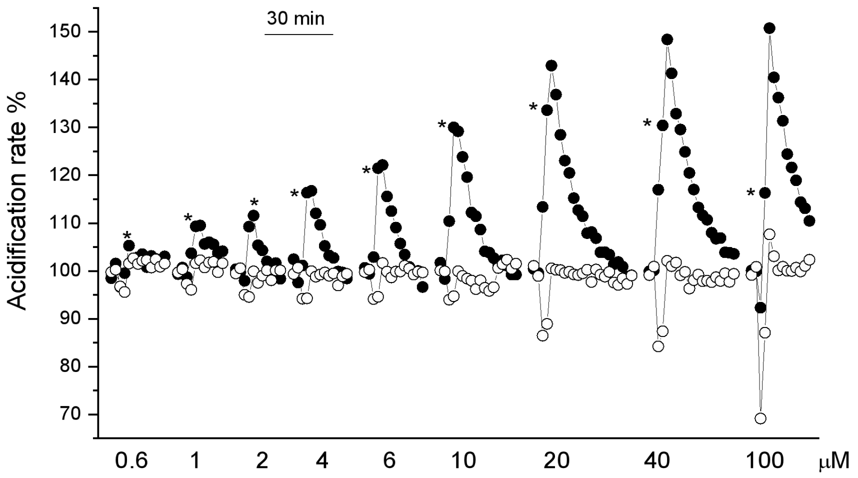

Supplementary Figure 4. Time-dependent ECAR of NIH-MDR-G185 (●) and NIH-3T3 (wild type) (○)cells induced by increasing concentrations of dibucaine normalized to the basal ECAR at time = 0, defined as 100%. Cells in MEM medium were exposed to two subsequent dibucaine injections in each measurement cycle. The stars* indicate the second stimulation point where steady state ATP hydrolysis rates can be reached. After this point the drug was flushed out with drug-free medium until basal activity was reached again. Notably, upon flushing out the second, inhibitory dibucaine molecule, the activity increases strongly at the three highest concentrations. The down peaks at the injection points reflect the cellular the transient increase in pH caused by the injection of the concentrated cationic drug solution. It is less pronounced in NIH-MDR-G185 than in wild type cells. Analogous Figures are shown in ref.[1].

REFERENCES

1. Gatlik-Landwojtowicz E, Aanismaa P, Seelig A. Quantification and characterization of P-glycoprotein-substrate interactions. *Biochemistry* 2006;45:3020-32. [DOI: 10.1021/bi051380+]

2. Gerebtzoff G, Li-Blatter X, Fischer H, Frentzel A, Seelig A. Halogenation of drugs enhances membrane binding and permeation. *Chembiochem* 2004;5:676-84. [DOI: 10.1002/cbic.200400017]

3. Seelig A, Blatter XL, Frentzel A, Isenberg G. Phospholipid binding of synthetic talin peptides provides evidence for an intrinsic membrane anchor of talin. *J Biol Chem* 2000;275:17954-61. [DOI: 10.1074/jbc.M002264200]

4. Seelig A, Landwojtowicz E, Fischer H, Li Blatter X. Towards P-glycoprotein structure-activity relationships. In: Waterbeemd vd, Lennernäs, Artursson, editors. Drug bioavailability/estimation of solubility, permeability and absorption. Weinheim: Wiley/VCH; 2004. pp. 461-92.

5. Litman T, Nielsen D, Skovsgaard T, Zeuthen T, Stein WD. ATPase activity of P-glycoprotein related to emergence of drug resistance in Ehrlich ascites tumor cell lines. *Biochim Biophys Acta* 1997;1361:147-58. [DOI: 10.1016/s0925-4439(97)00025-2]

6. Bruggemann EP, Currier SJ, Gottesman MM, Pastan I. Characterization of the azidopine and vinblastine binding site of P-glycoprotein. *J Biol Chem* 1992;267:21020-6. [DOI: PMID: 1356986]

7. Aanismaa P, Seelig A. P-glycoprotein kinetics measured in plasma membrane vesicles and living cells. *Biochemistry* 2007;46:3394-404. [DOI: 10.1021/bi0619526]

8. Nervi P, Li-Blatter X, Aanismaa P, Seelig A. P-glycoprotein substrate transport assessed by comparing cellular and vesicular ATPase activity. *Biochim Biophys Acta* 2010;1798:515-25. [DOI: 10.1016/j.bbamem.2009.11.022]

9. Hegner D. Age-dependence of molecular and functional changes in biological membrane properties. *Mech Ageing Dev* 1980;14:101-18. [DOI: 10.1016/0047-6374(80)90109-8]

10. Heerklotz H, Seelig J. Titration calorimetry of surfactant-membrane partitioning and membrane solubilization. *Biochim Biophys Acta* 2000;1508:69-85. [DOI: 10.1016/s0304-4157(00)00009-5]

11. Li-Blatter X, Beck A, Seelig A. P-glycoprotein-ATPase modulation: the molecular mechanisms. *Biophys J* 2012;102:1383-93. [DOI: 10.1016/j.bpj.2012.02.018]

12. Seelig A. Local anesthetics and pressure: a comparison of dibucaine binding to lipid monolayers and bilayers. *Biochim Biophys Acta* 1987;899:196-204. [DOI: 10.1016/0309-1651(90)91206-j]

13. Litman T, Zeuthen T, Skovsgaard T, Stein WD. Structure-activity relationships of P-glycoprotein interacting drugs: kinetic characterization of their effects on ATPase activity. *Biochim Biophys Acta* 1997;1361:159-68. [DOI: 10.1016/s0925-4439(97)00026-4]

14. Li-Blatter X, Seelig A. Exploring the P-glycoprotein binding cavity with polyoxyethylene alkyl ethers. *Biophys J* 2010;99:3589-98. [DOI: 10.1016/j.bpj.2010.10.033]

15. Meier M, Blatter XL, Seelig A, Seelig J. Interaction of verapamil with lipid membranes and P-glycoprotein: connecting thermodynamics and membrane structure with functional activity. *Biophys J* 2006;91:2943-55. [DOI: 10.1529/biophysj.106.089581]

16. McConnell HM, Owicki JC, Parce JW et al. The cytosensor microphysiometer: biological applications of silicon technology. *Science* 1992;257:1906-12. [DOI: 10.1126/science.1329199]

17. Zwick M, Esposito C, Hellstern M, Seelig A. How phosphorylation and ATPase activity regulate anion flux though the cystic fibrosis transmembrane conductance regulator (CFTR). *J Biol Chem* 2016;291:14483-98. [DOI: 10.1074/jbc.M116.721415]

18. Dawson RJ, Locher KP. Structure of a bacterial multidrug ABC transporter. *Nature* 2006;443:180-5. [DOI: 10.1038/nature05155]

19. Dawson RJ, Locher KP. Structure of the multidrug ABC transporter Sav1866 from staphylococcus aureus in complex with AMP-PNP. *FEBS Lett* 2007;581:935-8. [DOI: 10.1016/j.febslet.2007.01.073]

20. Xu Y, Seelig A, Bernèche S. Unidirectional transport mechanism in an ATP dependent exporter. *ACS Cent Sci* 2017. [DOI: 10.1021/acscentsci.7b00068]

21. Xu Y, Seelig A, Berneche S. Unidirectional transport mechanism in an ATP dependent exporter. *ACS Cent Sci* 2017;3:250-8. [DOI: 10.1021/acscentsci.7b00068]

22. Ataullakhanov FI, Vitvitsky VM. What determines the intracellular ATP concentration. *Biosci Rep* 2002;22:501-11. [DOI: Doi 10.1023/A:1022069718709]

23. Beis I, Newsholme EA. The contents of adenine nucleotides, phosphagens and some glycolytic intermediates in resting muscles from vertebrates and invertebrates. *Biochem J* 1975;152:23-32. [DOI: 10.1042/bj1520023]

24. Sharom FJ, Liu R, Qu Q, Romsicki Y. Exploring the structure and function of the P-glycoprotein multidrug transporter using fluorescence spectroscopic tools. *Semin Cell Dev Biol* 2001;12:257-65. [DOI: 10.1006/scdb.2000.0251]

25. Beck A, Aanismaa P, Li-Blatter X, Dawson R, Locher K, Seelig A. Sav1866 from staphylococcus aureus and P-glycoprotein: similarities and differences in ATPase activity assessed with detergents as allocrites. *Biochemistry* 2013;52:3297-309. [DOI: 10.1021/bi400203d]

26. Choudhury HG, Tong Z, Mathavan I et al. Structure of an antibacterial peptide ATP-binding cassette transporter in a novel outward occluded state. *Proc Natl Acad Sci USA* 2014;111:9145-50. [DOI: 10.1073/pnas.1320506111]

27. Agboh K, Lau CHF, Khoo YSK et al. Powering the ABC multidrug exporter LmrA: how nucleotides embrace the ion-motive force. *Sci Adv* 2018;4:eaas9365. [DOI: 10.1126/sciadv.aas9365]

28. Thomas C, Aller SG, Beis K et al. Structural and functional diversity calls for a new classification of ABC transporters. *FEBS Lett* 2020;594:3767-75. [DOI: 10.1002/1873-3468.13935]

29. Szollosi D, Rose-Sperling D, Hellmich UA, Stockner T. Comparison of mechanistic transport cycle models of ABC exporters. *Biochim Biophys Acta Biomembr* 2018;1860:818-32. [DOI: 10.1016/j.bbamem.2017.10.028]

30. Qu Q, Russell PL, Sharom FJ. Stoichiometry and affinity of nucleotide binding to

P-glycoprotein during the catalytic cycle. *Biochemistry* 2003;42:1170-7. [DOI: doi/10.1021/bi026555j]

31. Hellmich UA, Lyubenova S, Kaltenborn E et al. Probing the ATP hydrolysis cycle of the ABC multidrug transporter LmrA by pulsed EPR spectroscopy. *J Am Chem Soc* 2012;134:5857-62. [DOI: 10.1021/ja211007t]

32. Omote H, Al-Shawi MK. Interaction of transported drugs with the lipid bilayer and P-glycoprotein through a solvation exchange mechanism. *Biophys J* 2006;90:4046-59. [DOI: 10.1529/biophysj.105.077743]

33. Omote H, Al-Shawi MK. A novel electron paramagnetic resonance approach to determine the mechanism of drug transport by P-glycoprotein. *J Biol Chem* 2002;277:45688-94. [DOI: 10.1074/jbc.M206479200]

34. Xu Y, Egido E, Li-Blatter X et al. Allocrite sensing and binding by the breast cancer resistance protein (ABCG2) and P-glycoprotein (ABCB1). *Biochemistry* 2015;54:6195-206. [DOI: 10.1021/acs.biochem.5b00649]

35. Seelig A. A general pattern for substrate recognition by P-glycoprotein. *Eur J Biochem* 1998;251:252-61. [DOI: 10.1046/j.1432-1327.1998.2510252.x.]

36. Litman T, Zeuthen T, Skovsgaard T, Stein WD. Competitive, non-competitive and cooperative interactions between substrates of P-glycoprotein as measured by its ATPase activity. *Biochim Biophys Acta* 1997;1361:169-76. [DOI: 10.1016/s0925-4439(97)00027-6]
